# Comparative metabolomic profiling of *Arabidopsis thaliana* roots and leaves reveals complex response mechanisms induced by a seaweed extract

**DOI:** 10.3389/fpls.2023.1114172

**Published:** 2023-03-09

**Authors:** Thi Linh Chi Tran, Damien L. Callahan, Md Tohidul Islam, Yichao Wang, Tony Arioli, David Cahill

**Affiliations:** ^1^ School of Life and Environmental Sciences, Deakin University, Geelong, VIC, Australia; ^2^ School of Life and Environmental Sciences, Centre for Cellular and Molecular Biology, Deakin University, Burwood, VIC, Australia; ^3^ Seasol International R&D Department, Bayswater, VIC, Australia

**Keywords:** *Durvillaea potatorum*, *Ascophyllum nodosum*, *Arabidopsis thaliana*, metabolomics, biostimulants

## Abstract

Seaweed extracts are a prominent class of biostimulants that enhance plant health and tolerance to biotic and abiotic stresses due to their unique bioactive components. However, the mechanisms of action of biostimulants are still unknown. Here, we have used a metabolomic approach, a UHPLC-MS method, to uncover the mechanisms induced following application to *Arabidopsis thaliana* of a seaweed extract derived from *Durvillaea potatorum* and *Ascophyllum nodosum*. We have identified, following the application of the extract, key metabolites and systemic responses in roots and leaves across 3 timepoints (0, 3, 5 days). Significant alterations in metabolite accumulation or reduction were found for those belonging to broad groups of compounds such as lipids, amino acids, and phytohormones; and secondary metabolites such as phenylpropanoids, glucosinolates, and organic acids. Strong accumulations of TCA cycle and N-containing and defensive metabolites such as glucosinolates were also found revealing the enhancement of carbon and nitrogen metabolism and defence systems. Our study has demonstrated that application of seaweed extract dramatically altered the metabolomic profiles of *Arabidopsis* and revealed differences in roots and leaves that varied across the timepoints tested. We also show clear evidence of systemic responses that were initiated in the roots and resulted in metabolic alterations in the leaves. Collectively, our results suggest that this seaweed extract promotes plant growth and activates defence systems by altering various physiological processes at the individual metabolite level.

## 1 Introduction

The agriculture sector is currently being challenged to improve crop productivity, efficiently use nutrient resources and to provide high-quality crop products that will provide sufficient food for a fast-growing global population under a climate-changed environment. Biostimulants, especially those that are plant-based, have emerged as a potentially sustainable and ecofriendly solution for the agriculture sector due to their bioactive components which have been shown through many studies to enhance crop growth and stress tolerance (Shukla et al., 2019; Ali et al., 2021; Shukla et al., 2021). Biostimulants are divided into different categories:(1) fluvic and humic acids, (2) protein hydrolysates and amino acids, (3) seaweed extracts, (4) microbial inoculants and (5) plant extracts and silicon (Goni et al., 2016; Rouphael et al., 2020). Among these different types of biostimulants, seaweed extracts have gained increased attention. It has been widely reported, for example, that seaweed extracts enhance overall plant health and productivity by improving seed germination (Rayorath et al., 2007); photosynthesis and root development (Alam et al., 2014); flowering and fruit set (Dookie et al., 2021); and also by enhancing fruit taste and quality (Trejo Valencia et al., 2018). It has also been demonstrated that seaweed extracts alleviate the negative effects caused by abiotic stress (Goni et al., 2018; Rasul et al., 2021) and biotic stress (Frioni et al., 2019; Shukla et al., 2019).

The biostimulant actions of seaweed extracts have primarily been associated with stimulation of the jasmonic acid, salicylic acid, and ethylene-mediated defence pathways, which trigger the up regulation of well-characterised defence- and stress-related genes such as PR (*pathogen related)*, MAPK (*mitogen activated protein kinase*) genes and WRKYs (WRKY domains of the transcription factors) that results in accumulation of defence metabolites such as antioxidants, phenylpropanoids and fatty acids (Tugizimana et al., 2018; De Saeger et al., 2019; Shukla et al., 2021). Despite the reportedly significant benefits of seaweed extracts on plants, their specific effects and underlying mechanisms are broadly dependent on the diverse compositions of the extracts, the application procedure (rate, time, and frequency) and the plant species to which they are applied (Ali et al., 2021). Therefore, an in-depth investigation of molecular metabolism is required to elucidate which biochemical and biophysiological process are involved in the response to biostimulants and the resultant effects on plants. This understanding will facilitate their most efficient and accurate application for optimization of plant growth and yield.

‘Omics’ approaches, especially that of metabolomics, have become a powerful method to provide signatures of altered metabolites that can be directly linked to the biological status of plant organs, tissues, and cells under a variety of growth conditions. Ultra high-pressure liquid chromatography (UHPLC) combined with high resolution mass spectrometry (MS) is now being employed as one of the most powerful mass spectrometry-based technologies available for detection and identification of metabolites, due to its highly sensitive and accurate mass detection and which uses advanced data processing to analyze greatly complex mixtures extracted from plant tissues (Gorrochategui et al., 2016). In particular, using an untargeted metabolomic approach allows comprehensive assessment of numerous compounds in a single biological sample (Patel et al., 2021). The untargeted approach is especially useful for providing an overview of changes in the plant metabolome under different conditions (Katam et al., 2022). For example, this technique has recently been successfully applied to investigate the metabolic changes induced by seaweed extracts from the kelp, *Ecklonia maxima* in corn (*Zea mays*) under drought stress (Tinte et al., 2022). Similarly, Omidbakhshfard et al. (2020) showed for Arabidopsis that was under severe oxidative stress the enhanced accumulation of a number of metabolites and alleviation of stress following treatment with a seaweed extract derived from *Ascophyllum nodosum.*

In previous research (Islam et al., 2021), we showed that application of an extract derived from *A. nodosum* and *D. potatorum* promoted the expression of a number of key stress- and priming-related genes in *Arabidopsis*. However, the underlying mechanisms by which the activation of these genes then affords protection against stress remain largely unknown. Hence, this current study was conducted to investigate, using an untargeted approach, the metabolomic profiles of *Arabidopsis* following application of seaweed extract to the roots. In addition to previous transcriptomic and gene expression data (Islam et al., 2020; Islam et al., 2021), we now provide a thorough interpretation of the complex relationships between metabolites and associated pathways with plant growth and their potential to provide resistance under adverse conditions. Such fundamental findings will contribute to developing biostimulants for sustainable use in agriculture.

## 2 Materials and methods

### 2.1 Plant material and growth conditions

Seeds of *Arabidopsis thaliana* ecotype Ler (LEHLE, Texas, USA, http://www.arabidopsis.com) were sterilized and grown following the method of Islam et al. (2020) and Islam et al. (2021). *Arabidopsis* seeds were sterilized in 1 mL of a solution containing 500 µL of ethanol 100% and 5 µL of H_2_O_2_ 30%, suspended in water agar 0.2% (w/v) and stratified at 4°C for two days. The seeds were then sown into 9-cm Petri plates that contained Murashige and Skoog (MS) medium made with 0.8% (w/v) bacteriological agar (pH 5.7). The plates were placed into a growth chamber with 16: 8 h (light: dark photoperiod), light intensity of 100 μmol photons m^−2^ s ^−1^, at 21 ± 2°C for 14 days.

After 14 days, uniform-sized seedlings were selected for transferral into sand (Bunnings, Waurn Ponds, Australia) within a 5 mL plastic disposable pipette tube (Axygen™, Blackburn, Australia) inserted with a piece of cotton wool (Woolworths, Waurn Ponds, Australia) to hold the sand in place. Prior to adding to the tube, the sand was autoclaved and then added to the tube that was filled within 0.5 cm of the top and then supplied with 1 mL of distilled water on the sand surface. A 1-cm-deep hole was then created by pushing another tube’s end into the sand. Individual *A. thaliana* plants were gently removed from the MS plates and carefully placed within the hole in the tube. A volume of 1 mL distilled water was further added to cover the roots with sand. Tubes were then vertically placed in a plastic holding rack and transferred to the growth chamber under the conditions described above.

### 2.2 Treatment of *Arabidopsis* plants with seaweed extract

The seaweed extract (SWE) used in this study was an alkaline hydrolysis product (Seasol, Bayswater, Australia) derived from two brown algae, *Durvillaea potatorum* and *Ascophyllum nodosum* for which detailed compositions of the extract have been previously reported (Wite et al., 2015). A dilution of 1:400 of SWE in distilled water was used for all treatments and was based on previous studies undertaken in the laboratory, field, and greenhouse (Mattner et al., 2013; Mattner et al., 2018; Islam et al., 2020; Arioli et al., 2021).

**Figure 1:**
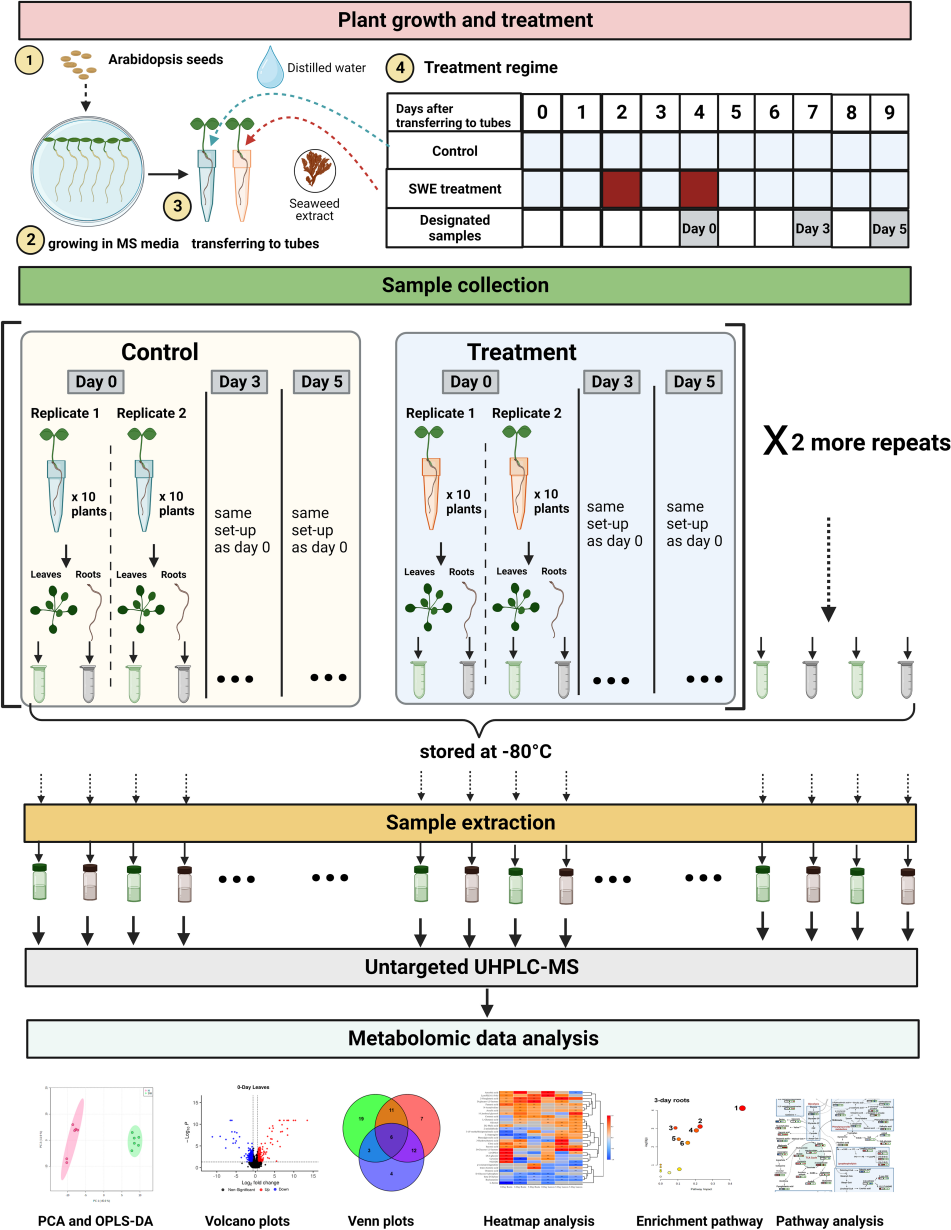
A summary of the methodology employed in this study. There were five major stages including plant growth and treatment, sample collection, sample extraction, untargeted UHPLC-MS, and metabolomic data analysis. For plant growth and treatment, *Arabidopsis thaliana* Col 0 seeds were surface-sterilized and grown in Murashige and Skoog (MS) media for 14 days. *Arabidopsis* seedlings were then transferred to 5 mL tubes containing sand. The control plants were provided with 700 µL of distilled water. The SWE-treated plants were provided with 700 µL of SWE (1:400 in distilled water) on the second and fourth day after transferring to the 5 mL tubes. Designated ‘day 0’, ‘day 3’ and ‘day 5’ samples were then harvested. Roots and leaves were separated from untreated and treated plants and were subsequently stored at -80°C. In total, there were 36 root samples and 36 leaf samples. Frozen samples were then extracted at the same time to provide materials for untargeted UHPLC-MS analysis. Metabolomic data were then analyzed using Compound Discoverer software version 3.3.1 and different R packages. The figure was generated using BioRender.

The SWE treatment regime was as follows: each day and up to nine days after transferring seedlings to 5 mL tubes, a control set of plants was provided with 700 µL of distilled water. A second set of plants was treated with 700 µL of SWE (1:400 in distilled water) on the second and fourth day after transplanting. One hour after the second SWE treatment on day 4, plants were harvested and designated as ‘day 0’ plants. To avoid damaging plants, tubes containing individual plants were submerged in distilled water to gently remove the root from the sand. Roots and leaves were then separated and briefly dried on absorbent paper. For each experimental repeat, two biological replicates of ‘day 0’ samples (replicate 1 and 2) were collected (**Figure 1**). For each replicate, ten control or SWE-treated plants were used. All ten root samples from the control or treated plants were then combined and placed in a 1.5 mL Eppendorf tube and then transferred to liquid nitrogen. Similar to roots, all ten control or treated leaf samples were combined, placed in a 1.5 mL Eppendorf tube and transferred to liquid nitrogen. All samples were subsequently stored at -80°C.

*Arabidopsis* plants were also collected at 3 days and 5 days after the second treatment with SWE and respectively designated as ‘day 3’ and ‘day 5’ samples. Root and leaf samples of day 3 and day 5 were harvested following exactly the same procedure as day 0 samples. The whole kinetics experiment was repeated three times, giving a total of 6 biological replicates of each control or SWE-treated root samples across three-time points. In total, therefore, there were 36 root samples, and 36 leaf samples. All samples were stored at -80°C before being used in the next stage: sample extraction.

### 2.3 Sample extraction

For each replicate, approximately 100 mg of frozen sample (stored at -80°C) of SWE-treated/untreated roots and leaves were used. Samples were weighted in 2 mL tubes containing acid-washed beads (Sigma Aldrich, Australia) and kept frozen in liquid nitrogen before being homogenized at 5 m/s × 30 s × 3 times in a FastPrep-24™ instrument (MP Biomedicals, Australia). A volume of 500 μL extraction solution containing a 2:3:3 ratio of water: acetonitrile: isopropanol (Sigma Aldrich, Australia) was added to the 2 mL tubes and again homogenized (5 m/s × 30 s × 3 times). Samples were then subjected to further centrifugation (13,300 g × 5 min) and the supernatants were then diluted to a 1:1 ratio with Milli-Q water. Finally, 150 µL of the diluted supernatant was transferred to 2 mL vials (Thermo Fisher Scientific, Australia) for the UHPLC-MS analysis. Two blank samples and two pooled biological quality control samples (PBQC) were prepared. The two blank samples contained only extraction solution and were used for the detection and identification of background compounds, which were subsequently removed. The two PBQC samples were created by mixing equally all individual samples together, and it was analyzed at the beginning and the end of the UHPLC-MS measurement (**Supplementary Figure S1**).

### 2.4 UHPLC-MS method

Samples were analysed following the method described by Silva-Campos et al. (2022) with some modifications. The UHPLC-MS system comprised a Vanquish Flex ultra high-pressure liquid chromatography system coupled with an OrbitrapExploris-240 high resolution mass spectrometer (MS; ThermoFisher Scientific). The liquid chromatography column was a 2.1 × 100 mm, 1.8 µm C18 Zorbax Elipse plus (Agilent), column temperature was 30°C, and mobile phase flow rate was 0.4 mL/min, with gradient elution. Mobile phase A was 0.1% formic acid in water and mobile phase B was 0.1% formic acid in acetonitrile. LC-MS grade solvents were used. The initial mobile phase composition was 5% B which was held for 1 min then linearly increased to 100% B over 9 min with a 2-min hold at 100% B then re-equilibration for 3 min at 2% B, giving a total run time of 15 min. The H-ESI source settings were: ion spray voltage 3800 V, sheath gas 50 (arb. units), sweep gas 1 arb, ion transfer tube 325°C, vaporizer temperature 350°C. A preliminary deep scan run was carried out using AcquireX workflow, which is an iterative data-dependent acquisition (DDA) strategy, in either positive (ESI+) and negative (ESI-) ionization modes. The deep-scan run collected full scan spectra at 60,000 resolution and MS/MS at 30,000 resolution (in both ESI+ and ESI-). Dynamic exclusion was set at 4 s. The deep scan MS/MS data were used for untargeted compound identification. For experimental samples, the instrument was subsequently operated in full scan mode with polarity switching at 60,000 resolution between 70-1050 m/z. Three normalized high energy collisional dissociation (HCD) MS/MS scans were used (20, 40, 80 NCE) and the MS/MS threshold was 5000 counts. The Easy-IC internal calibration was used in run-start mode giving sub-2 ppm mass accuracy.

### 2.5 Metabolomic data analysis

For data processing, raw data files collected by UHPLC-MS were imported into the software Compound Discoverer (CD) 3.3.1 (Thermo Fisher Scientific, USA). Data from both positive and negative ionization modes were analyzed. An untargeted workflow named “Untargeted Metabolomics with Statistics Detect Unknowns with ID using Online Databases and mzLogic” (Thermo Fisher Scientific, USA) with some modifications was applied for peak detection, deconvolution, alignment, and gap filling for the detection of unknown compounds. A full workflow and detailed settings are presented in **Supplementary Figure S2** and **Supplementary Table S1**. Briefly, selected spectra were aligned with mass tolerance (MT) < 5 ppm and maximum shift time was 2 min. Grouping of unknown compounds used a retention time (RT) tolerance < 0.5 min and MT< 5 ppm. Predicted composition node was also carried out using accurate mass, isotopic pattern, and MS/MS data with a mass tolerance window at 5 ppm. The metabolite identification was undertaken by searching against in-house and online databases, including: Mass List; ChemSpider (http://www.chemspider.com) used MS data (molecular weight or predicted formula) to search in 115 million chemical structures from 276 data sources; and mzCloud (https://www.mzcloud.org) that used MS/MS data to search against an online spectral fragmentation library of more than 9 million spectra. Consequently, compounds that were fully matched by predicted composition and/or mzCloud were selected to manually confirm structure using Fragment Ion Search (FISh) scoring. FISh scoring is an algorithm that compares the proposed fragments of compounds to the experimental fragmentation using the HighChem Fragmentation Library™ (Thermo Fisher Scientific, USA). In this study, metabolites were putatively identified to Metabolite Standard Initiative (MSI) level 2 (the similarity with MS data from public databases or literature) (Fiehn et al., 2007; Schymanski et al., 2014).

In addition, pathway analysis was performed using the Kyoto Encyclopedia of Genes and Genomes (KEGG), a database that contains more than 552 module pathways (https://www.genome.jp/kegg/pathway.html). The Human Metabolome Database (HMDB) with over 20 thousand compounds (many of which are plant-based compounds contained in the human diet) was used to classify different groups of compounds. Metabolites were functionally annotated by searching the Plant Metabolic Network database (https://plantcyc.org/) for *Arabidopsis thaliana* and previous publications in other plants.

Features detected from MS positive and negative ionization modes were combined and duplicated features were removed. Peak areas were normalized by using the constant median method. Normalized peak area data of unique features exported from CD 3.3.1 were formatted and uploaded to the MetaboAnalyst 5.0 [http://www.metaboanalyst.ca; Pang et al. (2021)] for Principal component analysis (PCA) and orthogonal projections to latent structures discriminant analysis (OPLS-DA) with log_10_ transformation. Significant pathway analysis was also carried out using the MetaboAnalyst 5.0. Significantly different metabolites were selected using the criteria of p < 0.05 (t-test, control vs treatment at each time point, unadjusted p-value) and log_2_ fold change (log_2_FC) > 0.6 or < -0.6. The volcano diagram was visualized using EnhancedVolcano packages in R language. Venn and heatmap diagrams were generated using VennDiagram, and Heatmaply R packages, respectively. In this study, comparisons were considered between (1) SWE treatment and the control (2) root and leaves and (3) 0, 3, and 5 days after SWE treatment.

## 3 Results

### 3.1 Global metabolomic changes in leaves and roots following SWE treatment

To investigate the effects of SWE on *Arabidopsis*, metabolomic profiles of roots and leaves were investigated using a UHPLC-MS method. There was a range of features (from a minimum of 4107 up to 7640 features) detected in leaf and root samples at day 0, 3, and 5 after SWE root application.

A principal component analysis (PCA) score plot was generated to assess the potential sources of variability or bias in the data (**Supplementary Figure S3**). Despite using 3 independent batches of plants in this experiment, one limitation of this study is that there were two PBQC samples analysed (at the beginning and at the end of the analytical sequence). Therefore, to standardize untargeted profiling experiments using plant biostimulants, we recommend following guidelines for the use of quality control and system suitability samples in untargeted metabolomic studies, as described by Broadhurst et al. (2018) and Ghosson et al. (2018). For example, for an effective analytical sequence, multiple PBQC injections are required between each 5-6 test samples, and a tight clustering of PBQC samples represented on a PCA score plot is required to indicate an acceptable level of stability throughout the experiment.

**Figure 2:**
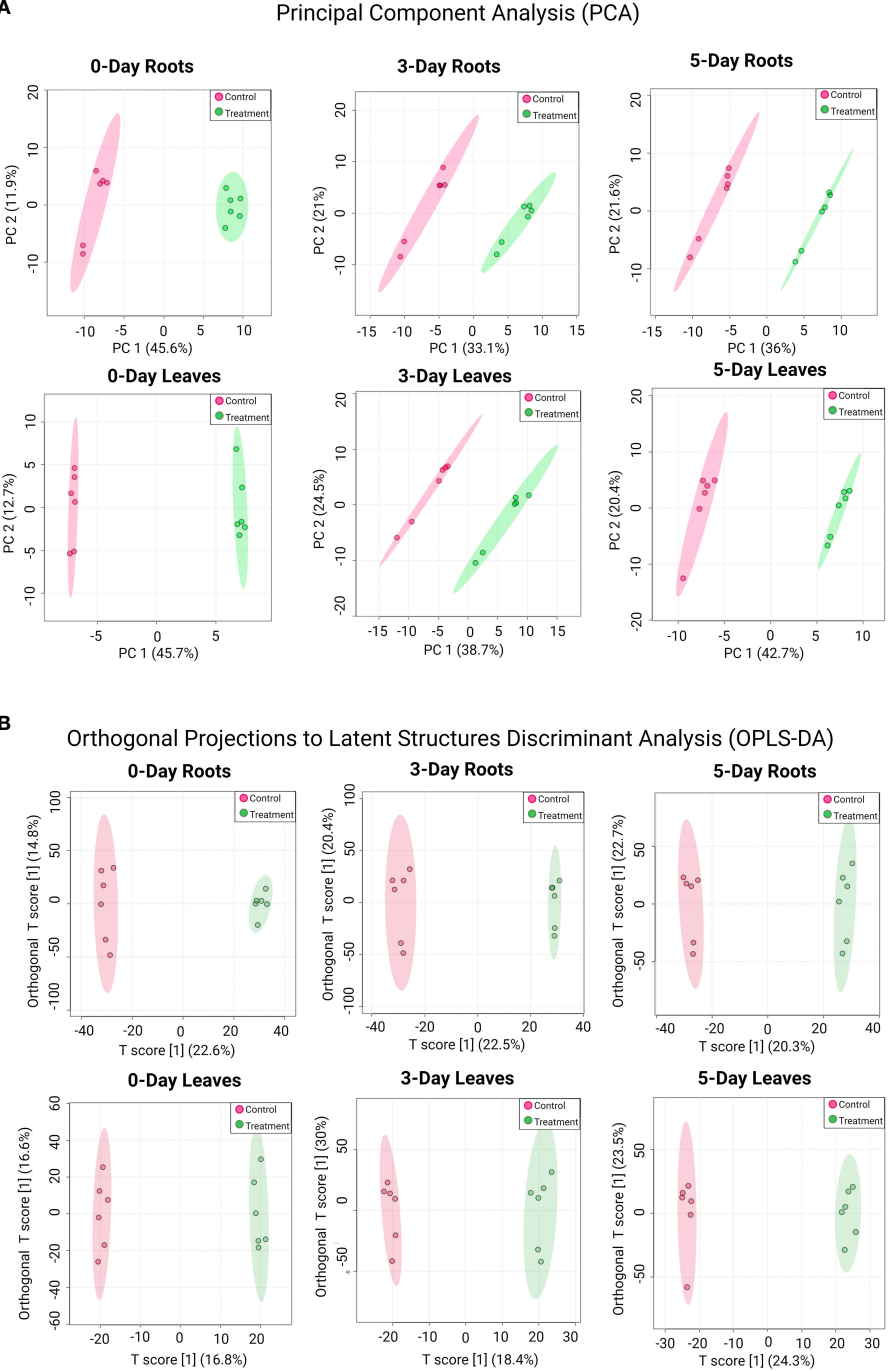
PCA **(A)** and OPLS-DA analysis **(B)** for detected compounds in SWE-treated and non-treated roots and leaves across different time points. Data were transformed by log_10_. Different treatment groups are indicated by the color (red color represents control samples and green color represents SWE-treated samples). Data presented are from six replicates with three individual experimental repeats.

PCA plots were also generated to determine similarities and differences between the control and treated samples in roots or leaves at each time point (**Figure 2A**). In all cases, samples from the SWE-treated groups were clustered together and separated from the control groups. An orthogonal projections to latent structures discriminant analysis (OPLS-DA) was subsequently performed to further highlight the dissimilarities between treatment groups (**Figure 2B**). Indeed, the analysis showed a clear separation between SWE-treated root/leaf samples and the control across all time points post treatment. Further, the robustness and performance of the model were evaluated using cross-validation and permutation tests (**Supplementary Figure S4**). For the cross-validation test, in all cases, R2Y was above 90%, and the Q2 of the “p” component was higher than 70%. The permutation test showed that p-value was less than 0.05. These results indicated that the discrimination of the two groups is significant and reliable. Therefore, there was a significant reprogramming of the metabolome in both leaves and roots following the application of SWE.

It should be noted here that the SWE used in this study contains several major compounds, including laminarins, auxin, cytokinin and betaines, which are present at relatively low concentrations in diluted SWE. For example, the concentration of auxin and cytokinin were 0.06 and 0.0013 µg/L in a 1:200 diluted SWE (Wite et al., 2015). In our study, we used an even lower concentration of SWE (1:400 dilution), which would have contributed a negligible amount of these compounds to the treated plants and were, consequently, not detected in our analysis.

**Figure 3:**
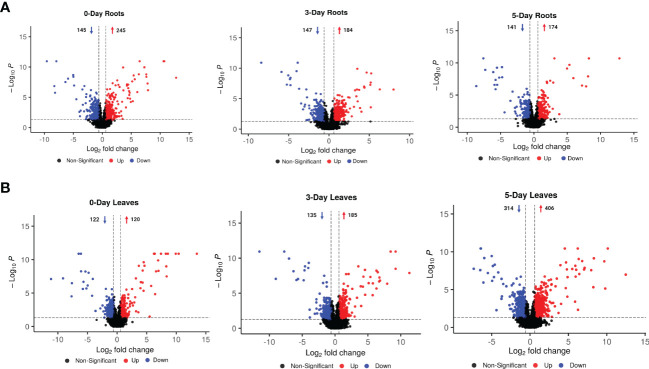
Volcano diagram analysis for metabolites that were significantly changed in accumulation and were filtered by using the criteria of p-value < 0.05 and log_2_FC > 0.6 or < -0.6 (between SWE-treated and non-treated samples in the same tissue at one time point) in 0-, 3- and 5-day roots **(A)** and leaves **(B)**. Red and blue color represent the increased and decreased accumulation of metabolites, respectively. The arrows and numbers represent the number of metabolites that were increased and decreased in accumulation, respectively. Data presented are from six replicates with three individual experimental repeats.

Volcano diagram analysis was performed to determine the number of compounds that significantly accumulated or were reduced (p-value < 0.05 and log_2_FC > 0.6 or < -0.6) in leaves and roots after 0, 3, or 5 days of SWE treatment (**Figure 3**). In most cases, the number of compounds that were significantly accumulated were higher than those that were reduced in accumulation. In roots, a greater number of metabolites that were differentially changed in relative abundance was observed at day 0 compared with day 3 and day 5 (174 were accumulated and 141 were reduced in abundance) (**Figure 3A**). This indicated a clear initial response and a change at the metabolomic level for plants after two root applications of SWE at day 0. In leaves, the highest number of differential compounds were at day 5 (405 were accumulated and 314 were reduced) (**Figure 3B**), implying a remarkable reprogramming toward accumulation post-SWE treatment.

Identification of those metabolites that significantly changed in abundance (p-value < 0.05 and log_2_FC > 0.6 or < -0.6) was confirmed by using the predicted composition and/or mzCloud matching in combination with FISh scoring. As a result, 89 compounds in roots and leaves at different time points were filtered for further analysis. Detailed information including classification and references of these compounds is given in **Supplementary Tables S2**, **S3**.

### 3.2 Specific changes of metabolites in roots and leaves after SWE treatment

**Figure 4:**
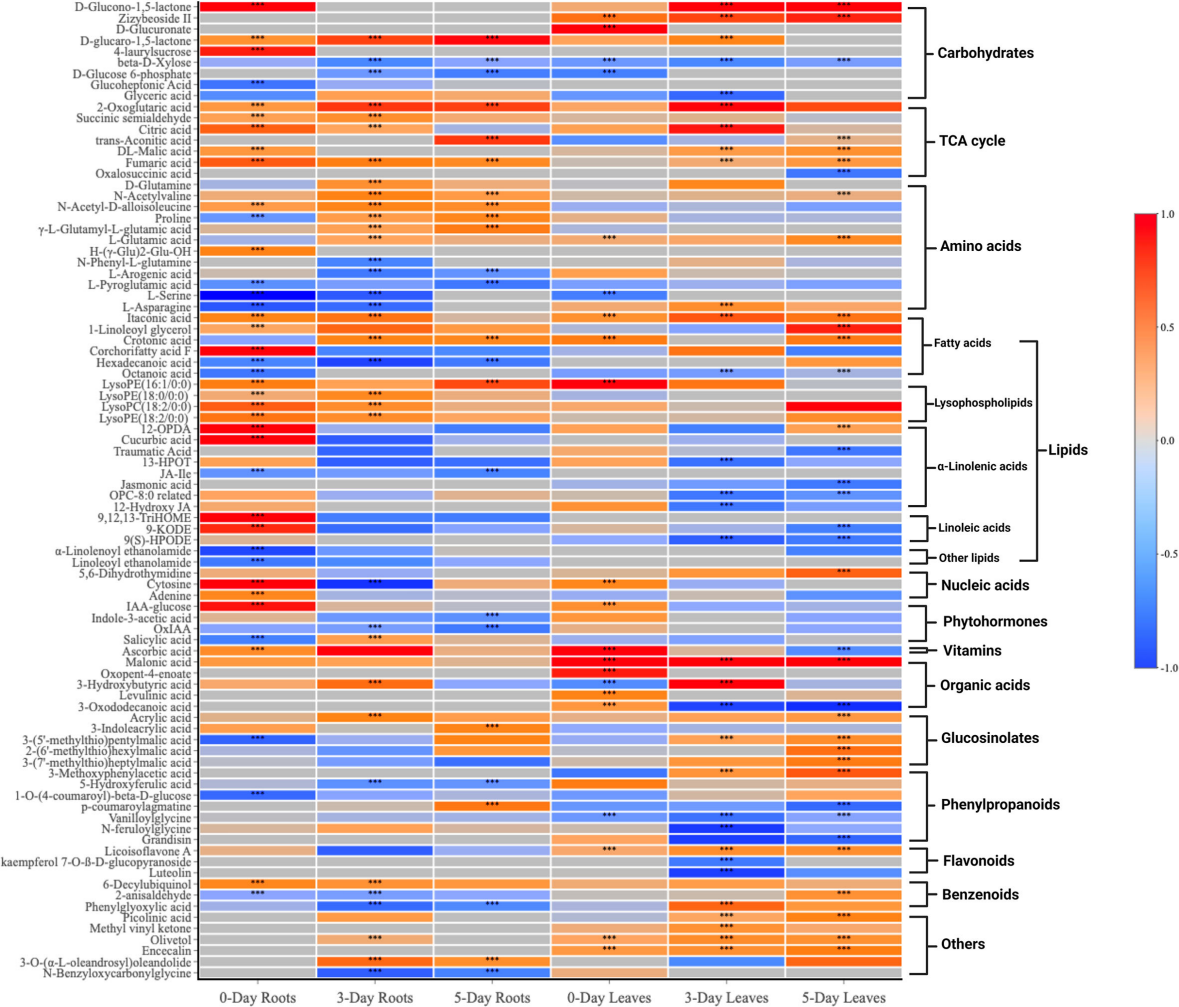
A heatmap of 89 compounds of interest and their classification in both roots and leaves at 0, 3, 5 days after SWE treatment. Red and blue color represent the increased or reduced accumulation of metabolites respectively. The asterisks indicate significant differences (t-test and p-value <0.05) between the treated and the control samples.

The 89 compounds were classified into different groups using the HMDB database. These compounds included primary metabolites: lipids and derivatives (23 in number), amino acids and derivatives (12), carbohydrates and derivatives (9), TCA cycle (7), hormones and derivatives (4), nucleotides and derivatives (3), and vitamins (1); and secondary metabolites: phenylpropanoids and derivatives (7), glucosinolates and derivatives (5), organic acids (5), flavonoids (3), benzenoids (3), alkaloids (1) and others (6) (**Figure 4** and **Supplementary Table S2**).

**Figure 5:**
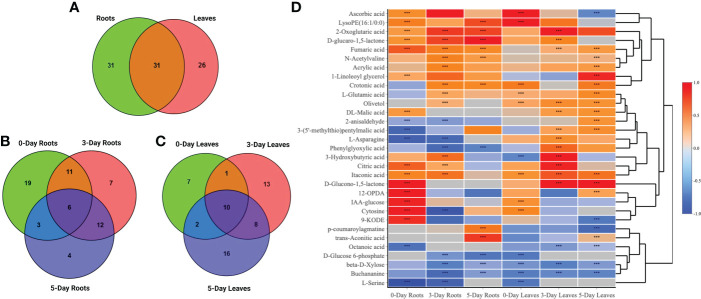
Venn diagrams and heatmap analysis. Venn diagrams showed commonalities and differences among changed metabolites in roots and leaves **(A)**, in roots **(B)** or leaves only **(C)** after 0, 3, and 5 days treated with the SWE. **(D)** A heatmap of 31 commonly identified compounds in both roots and leaf samples. The asterisks indicate significant differences (t-test and p-value <0.05) between the treated and the control samples.

Venn diagrams were generated to compare compounds detected in roots versus leaves (**Figure 5A**) and between 0-day, 3-day and 5-day samples within the same root (**Figure 5B**) or leaf tissues (**Figure 5C**). A higher number of metabolites that were differentially accumulated were identified in the roots (62) compared to the leaves (57) (**Figure 5A**). There were 31 compounds that showed significant changes in accumulation in both leaves and roots, while 31 and 26 compounds were uniquely identified in roots and leaves, respectively. A large proportion of the common compounds showed a similar pattern of change in that 17 and 5 compounds were found to be accumulated or reduced in relative abundance, respectively, in both leaves and roots at different time points (**Figure 5D**). When considered at the same harvest day, several metabolites showed reduction in the roots but accumulation in the leaves or vice versa, accumulation in the roots and reduction in the leaves. For example, asparagine and phenylglyoxylic acid were reduced at 3 days in the roots but accumulated in the leaves at the same time point. P-coumaroylagmatine showed the opposite at 5 days where it accumulated in the roots but was reduced in the leaves. At day 0, LysoPE (16:1/0:0), cytosine, IAA-glucose showed higher relative content in both parts whilst serine was the only compound that showed remarkably decreased accumulation (> -1.5-fold) in both leaves and roots. Citric acid, 2-oxoglutaric acid, crotonic acid, aconitic acid and D-glucaro-1,5-lactone were significantly increased in accumulation at either day 3 or day 5. Notably, fumaric acid and itaconic acid showed higher accumulation in leaves and roots from day 0 to day 5 which contrasted, for example, to the decreased accumulation of ß-D-Xylose across the three time points (**Figure 5D**).

**Figure 6:**
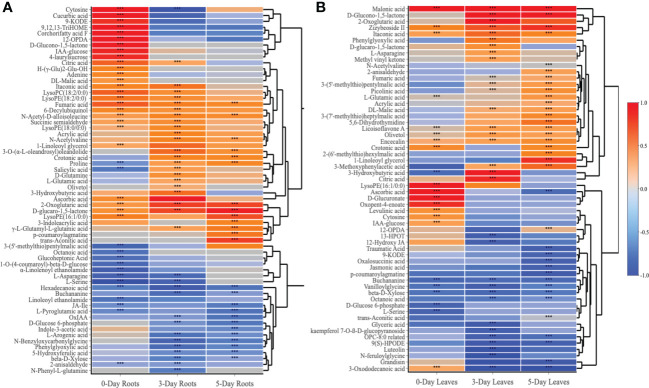
Heatmap analysis of metabolites that altered in roots **(A)** and leaves **(B)** at day 0, day 3 and day 5 following the SWE treatment. Log_2_FC data were used to represent the up (red color) or down (blue color) accumulation of compounds. The asterisks indicate significant differences (t-test and p-value <0.05) between the treated and the control samples.

When comparing the 89 compounds in leaves and roots, a higher number of secondary metabolites, including phenylpropanoids (5), organic acids (5), glucosinolates (4), and flavonoids (3) were detected in the leaves, whereas the number of amino acids (15) and hormones (4) were greater in the roots (**Figure 6** and **Supplementary Tables S4**, **S5**). Lipids, TCA-cycle compounds and carbohydrates were similar in number between both roots and leaves. In roots, out of 62 compounds that differentially accumulated, the highest number of identified compounds (39) was observed at day 0 when compared to day 3 and day 5 (**Figure 5B**). The number of compounds that were unique at day 0 was 19, 7 at day 3 and 4 at day 5, while only 6 metabolites were significantly changed in accumulation across all three time points. At day 0 in the roots, 26 and 13 metabolites were increased and decreased in accumulation respectively, whereas those numbers for compounds at day 3 were 22 and 14, and 13 and 12 compounds for day 5 respectively (**Supplementary Table S4**). For common metabolites in roots, N-Acetyl-D-alloisoleucine, D-glucaro-1,5-lactone, fumaric acid and 2-Oxoglutaric acid were significantly increased when only hexadecanoic acid showed reduced accumulation across all time points (**Figure 6A** and **Supplementary Table S6**). Various metabolites were significantly accumulated at day 3 and 5 but showed decrease in accumulation at day 0 such as proline, or showed no changes in relative abundance at day 0 such as crotonic acid, γ-L-Glutamyl-L-glutamic acid, N-Acetylvaline and 3-O-(α-L-oleandrosyl)oleandolide. Different lipid metabolites were specifically increased in their relative content at day 0 compared to day 3 and day 5 in the roots such as 1-linoleoyl glycerol, corchorifatty acid F, 12-OPDA, 9,12,13-TriHOME, and 9-KODE.

In contrast to the root samples, out of 57 compounds, the highest number of metabolites that were significantly changed in accumulation was observed at day 5 in the leaves with 36 detected compounds (22 showed an increase in accumulation and 14 decreased in accumulation respectively) (**Figure 5C** and **Supplementary Table S5**). In leaves, in comparison to the roots, there were more common metabolites (10 in number) at all three harvested time points, whereas 8, 13 and 16 compounds were uniquely present in the leaves at day 0, day 3 or day 5 respectively. For common compounds across all three time points in the leaves, malonic acid, licoisoflavone A, encecalin and zizybeoside II showed an increase in the relative content, while vanilloylglycine showed a decrease in accumulation (**Supplementary Table S7**). At day 3 and day 5, malic acid and picolinic acid showed higher relative abundance but no change at day 0 in the leaves, in contrast to the decrease in relative content of lipid-related compounds (octanoic acid, OPC-8:0 derivatives and 9(S)-HPODE) (**Figure 6B**). Out of 16 unique metabolites in 5-day leaves, lipids (5), glucosinolates (3), phenylpropanoids (2) and TCA cycle (2) were major groups of compounds that were significantly changed in accumulation (**Figure 6B** and **Supplementary Table S5**). Several compounds were strongly increased (fold change > 1.5) in accumulation in 5-day leaf samples such as 3-(7’-methylthio)heptylmalic acid, 2-(6’-methylthio)hexylmalic acid and 1-linoleoyl glycerol, while jasmonic acid and traumatic acid were decreased in accumulation.

### 3.3 Specific changes of pathways in roots and leaves after SWE treatments

**Figure 7:**
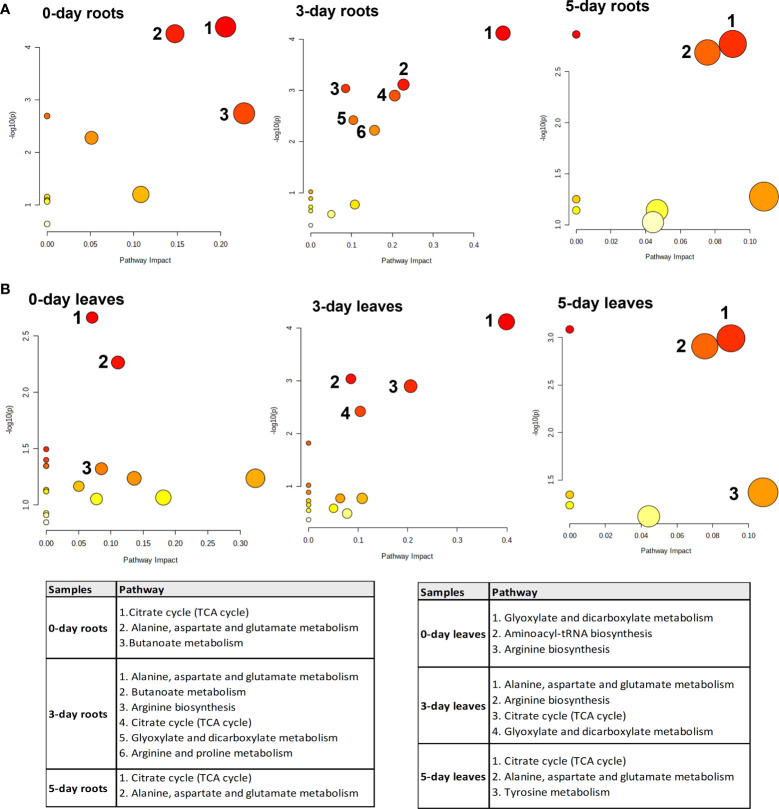
Significant enrichment pathway analysis of metabolites which showed accumulation in roots **(A)** and leaves **(B)** after the application of SWE. The y-axis (-log (p)) indicates the p-value of the pathway enrichment analysis, represented by the bubble color (darker color means more significant enrichment). The x-axis is the impact factor of topological analysis, represented by the bubble size (bigger bubble means a higher impact).

**Figure 8:**
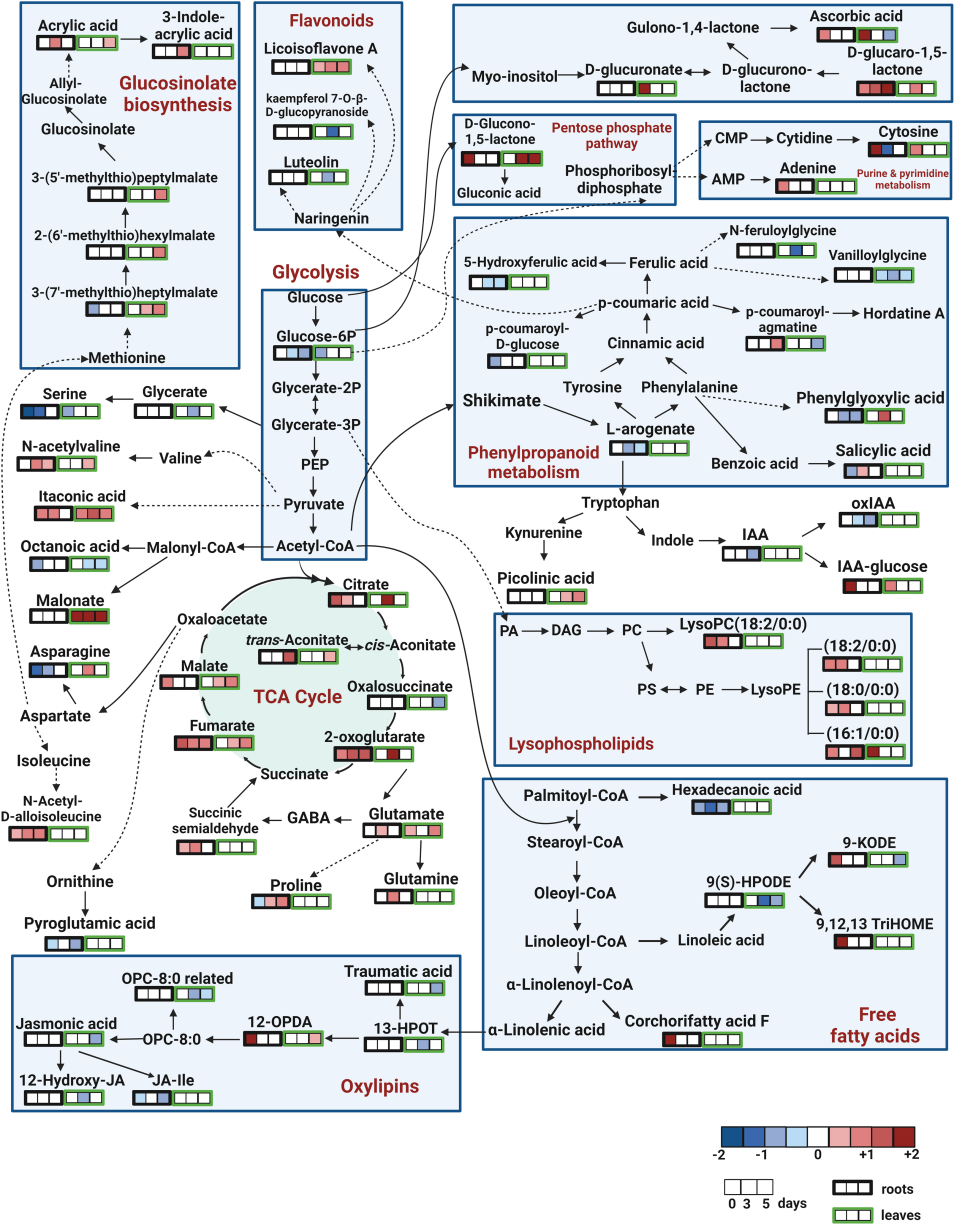
Pathway analysis of metabolites that altered in roots and leaves at day 0, day 3 and day 5 following SWE treatment. Out of 89 compounds of interest, 62 compounds were found in the KEGG pathway database, which was used to create this figure. The remaining 27 compounds are not shown in the figure. Log_2_FC data were used to represent the accumulation of the compounds (red color), or reduced accumulation (blue color), or no statistical change (white) of compounds.

To identify the biological pathways that were significantly altered by the treatment with SWE, the KEGG database combined with Metaboanalyst 5.0 were used. **Figure 7** presents those pathways that were statistically significant (p-value < 0.05) and with high impact generated from only metabolites that were accumulated in leaves and roots (derived from the list of 89 important compounds) at day 0, day 3 and day 5 after SWE treatment. It is clearly shown that the TCA cycle and alanine, asparagine, and glutamine pathways were the most common pathways that were up regulated in both leaves and roots across day 0 to day 5 (except day 0 leaves). The KEGG pathway database was then further used to manually determine the connection between compounds that belong to the same metabolic pathways. We found that 62 of the 89 identified compounds were matched with a key metabolic pathway within the KEGG database (**Figure 8**). Most of the metabolites in the TCA cycle, such as malic acid, fumaric acid, 2-oxoglutaric acid, and citric acid, were strongly accumulated up to 5 days after SWE application in both roots and leaves. Apart from the TCA cycle, metabolites belonging to the glucose and pentose phosphate pathways, which contribute to carbon metabolism, also showed up regulation in the treated plants across the different time points. In addition, metabolites belonging to glucosinolate and lysophospholipid biosynthesis pathways were accumulated in leaves or roots of SWE-treated samples. In contrast, most compounds (except 12-OPDA) that contributed to the oxylipin biosynthesis pathway showed decreases in their relative abundance in the SWE-treated roots and leaves. Some metabolites of the phenylpropanoid and free fatty acid metabolic pathways showed either accumulation or reduction, indicating a complex regulatory system in the response of *Arabidopsis* to treatment with a seaweed-based biostimulant.

## 4 Discussion

It was widely reported that seaweed extracts contain various organic compounds such as polysaccharides, laminarin, ulvans, alginates, and galactans that can potentially enhance plant growth and development as well as trigger priming and resistance to stresses (Shukla et al., 2019; Ali et al., 2021; Tinte et al., 2022). Many commercial products derived from seaweed extracts have been developed and their effectiveness demonstrated (Shukla et al., 2019). Here, we investigated the potentially beneficial effects of two applications of a seaweed-based commercial product on *Arabidopsis* plants using a metabolomic approach. Reprogramming in metabolomic profiles were clearly shown in SWE-treated *Arabidopsis* leaves and roots. These metabolites included (1) primary compounds such as those of the TCA cycle, lipids, amino acids and carbohydrates and (2) secondary compounds such as glucosinolates, phenylpropanoids, and organic acids. Although differences existed between metabolomic profiles of root and leaves, many common up-accumulated metabolites were detected, which are directly involved in various fundamental growth and defence pathways. The mode of actions of SWE is likely to associate with induced priming and systemic mechanisms that have been demonstrated at the transcriptomic level in our previous studies (Islam et al., 2020; Islam et al., 2021).

### 4.1 Seaweed extracts induced alterations in primary metabolites related to growth and signaling

#### 4.1.1 The TCA cycle was the most up-regulated pathway in both leaves and roots

The TCA cycle is a series of reactions that generate energy by the oxidation of acetyl-CoA derived from carbohydrates, amino acids and fatty acids (Martinez-Reyes and Chandel, 2020). In the current study, we found the TCA cycle to be the most up-regulated pathway in SWE-treated plants, represented by the accumulation of major TCA cycle metabolites, including citric acid, aconitic acid, 2-oxoglutaric acid, fumaric acid, and malic acid (**Figure 8**). The increased accumulation of TCA cycle compounds has been observed in various plant species, including *Arabidopsis*, wheat, and maize following the application of biostimulants under normal conditions (Nephali et al., 2021), prior to herbicide-induced oxidative stress (Omidbakhshfard et al., 2020) or pathogen attack (Camanes et al., 2015). Citric acid can be transported to the cytosol to maintain the pH (Tahjib-Ul-Arif et al., 2021) or be utilized directly by the cell to promote the biosynthesis of lipid and nucleotides (Martinez-Reyes and Chandel, 2020). Citric acid was also demonstrated to be a scavenger and trigger defence responses in plants under stress conditions (Chele et al., 2021). Similarly, we found that accumulation of fumaric acid was greater in the roots compared to the leaves across time points. Fumaric acid has also been reported to accumulate with higher light intensities and with plant age in *Arabidopsis* (Chia et al., 2000). In addition, fumaric acid and malic acid also can become an alternative source of carbon for photosynthate and counteranions for nitrate (Nunes-Nesi et al., 2010). As shown by Schwachtje et al. (2018), these organic acids also can form the primary building blocks of defence-active compounds.

The TCA cycle may be also involved in both anabolism and catabolism and cycle intermediates can be transported from mitochondria to the cytosol to participate in metabolite biosynthesis, nitrate assimilation, photosynthesis, and photorespiration (Fernie et al., 2004; Araujo et al., 2012; Zhang and Fernie, 2018). For example, 2-oxoglutaric acid is a master regulator metabolite due to its role as a main carbon skeleton in nitrogen-assimilatory reactions. Indeed, 2-oxoglutaric acid participates in the synthesis of glutamic acid from ammonia following two pathways, (1) by direct reaction with ammonia *via* the catalyzation of glutamate dehydrogenase (GDH) or (2) combining with glutamine *via* the catalyzation of glutamate synthase (Huergo and Dixon, 2015). In our study, the increased relative abundance of 2-oxoglutaric acid may have led to the higher accumulation of glutamic acid, glutamine, and proline at 3 and 5 days after SWE treatment. A recent study by Gai et al. (2022) has shown that foliar application of 2-oxoglutaric acid significantly increased the content of glutamic acid, glutamine and proline by enhancing the activation of glutamine synthetase and glutamate dehydrogenase in soybean under drought stress.

Taken together, the increased accumulation of TCA cycle metabolites and N-containing amino acids indicate that carbon and nitrogen metabolism were greatly activated in the *Arabidopsis* plants after treatment with seaweed extract. The enhancement of the TCA cycle up to 5 days after SWE treatment may provide more energy and precursors for plant growth improvement and defence against pathogens.

#### 4.1.2 Lipids, amino acids, carbohydrates and other primary metabolites related to growth and development and stress responses were accumulated following SWE treatment

Lipids are major components of cell membranes and are a source of signaling compounds during stress responses (Okazaki and Saito, 2014; Barbaglia and Hoffmann-Benning, 2016). In our study, seaweed extract treatment significantly modified the composition of lipid profiles of both *Arabidopsis* leaves and roots with an enhancement of lysophospholipids and a decrease in oxylipins and free fatty acids. As suggested by Yu et al. (2021), remodeling of these lipids is considered as a strategy of acquiring resistance to stress conditions.

Enhanced accumulation of lysophospholipids (LPLs) were reported in the response to the application of biostimulants, salt and drought stress or wounding and pathogen attack (Hou et al., 2016). The accumulation of LPLs may result from the hydrolysis of fatty acids from phospholipids activated by phospholipase A (PLA) (Okazaki and Saito, 2014). In our study, one LPL, LysoPC (C18:2) accumulated at day 0 and day 3 in the *Arabidopsis* roots. Cho et al. (2012) also observed in tobacco plants that LysoPCs C18:2, C16:0 and C18:3 increased significantly following inoculation with the pathogen *Phytophthora nicotianae*. Additionally, LysoPCs were reported to have the ability to activate signal transducers such as Ca^2+^ flux, a tonoplast H^+^/Na^+^ antiporter and enhanced pH levels in cells, contributing to the production of phytoalexins (Viehweger et al., 2002). We also recorded higher amounts of LysoPEs, including LysoPE C18:2, C18:0 and C16:1 mostly in the root samples. Volz et al. (2021) reported that the application of LysoPE induces gene expression for SA biosynthesis and reactive oxygen species (ROS) signaling pathways, which consequently induced resistance of *Arabidopsis* following infection with the hemibiotrophic pathogen *Pseudomonas syringae*. In addition, we have shown that the relative abundance of itaconic acid was significantly increased in both SWE-treated leaves and roots across all three time points. Increased accumulation of itaconic acid was also recorded by Yang et al. (2019) in *Clematis terniflora* exposed to UVB irradiation and dark conditions. It has been proposed that itaconic acid plays a role as an antimicrobial metabolite due to its inhibitory effect on isocitrate lyase, a key enzyme in the glyoxylate pathway (a variation of the TCA cycle), contributing to limiting the growth of various pathogens (Cordes et al., 2015; Luan and Medzhitov, 2016).

However, we found that the relative abundance of free unsaturated fatty acids (UFAs) and related compounds including hexadecanoic acid, linoleic acid derivatives (9(S)-HPODE and 9-KODE), and oxylipins derived from α-linolenic acid were lower in the SWE-treated plants. Recent studies have reported the accumulation of essential UFAs such as linolenic, hexadecanoic, and stearic acid induced by biostimulants in tomato or maize (Othibeng et al., 2021; Rachidi et al., 2021). In our study, the phytohormone jasmonic acid (JA), JA derivatives (JA-Ile and 12-Hydroxy-JA) and intermediates in the oxylipin pathway (13-HPOT) were reduced. Oxylipin metabolites play an important role in the mediation of plant defence systems against pathogens and herbivores as well as in response to abiotic stress such as UV, drought and cold (Ruan et al., 2019; Ghorbel et al., 2021). However, under ambient conditions, JA and its derivatives were demonstrated to have negative impacts on plant growth, including inhibition of seedling growth, leaf expansion and primary root growth (Huang et al., 2017). Our study has indicated that JA-mediated pathways were repressed by SWE application and, therefore, may have reduced any negative impact on plant growth.

Many studies have demonstrated the important roles of amino acids in a range of metabolic processes in plants, including enzyme and protein structure, precursors of essential secondary metabolites, and signal transduction for plant growth and resistance against stress (Zeier, 2013; Alfosea-Simon et al., 2020). In the current study, glutamine, glutamic acid and its polypeptides (γ-L-Glutamyl-L-glutamic acid and H-(γ-Glu)2-Glu-OH), N-acetylvaline, and proline showed increased content, while asparagine and serine decreased accumulation in the SWE treated roots and/or leaves across the three time points. Similar increases in abundance of amino acids, including proline, glutamine, and γ-L-Glutamyl-L-glutamic acid were reported by Yang et al. (2020) for *Puccinellia tenuiflora* inoculated with a microbial biostimulant under salt stress. The accumulation of amino acids in such systems may alter the osmotic status of the plants to maintain membrane stability, therefore improving salt tolerance (Shulaev et al., 2008). For example, proline is a well-known and essential compatible osmolyte accumulating in plants under adverse conditions (Verbruggen and Hermans, 2008; Lehmann et al., 2010). Proline may reduce ROS production, enhance protein stabilization processes and contributes to stress signaling pathways (Szabados and Savoure, 2010; Khan et al., 2019). In our study, proline was reduced in the leaves at day 0 but then significantly increased at day 3 and day 5. Similarly, Goni et al. (2018) reported a higher concentration of proline in tomato plants treated with a seaweed-based biostimulant, allowing more effective water uptake into the plant body to avoid water shortage under drought stress. Glutamine and glutamic acid are common nitrogen-containing amino acids associated with transportation, storage, and recycling of nitrogen in green plant parts, germinating seeds and they support plant growth and responses to stress (Han et al., 2021). Forde and Lea (2007) showed that nitrogen from inorganic sources such as nitrates and ammonia can be converted to glutamic acid and glutamine. It was also shown that direct application of glutamic acid could increase carbon assimilation and enhance the content of glucose, isoleucine and proline, resulting in general growth improvement of tomato plants (Alfosea-Simon et al., 2020). Glutamine is generated from glutamic acid and ammonium *via* the catalyzation of glutamine synthetase to rapidly induce expression of the key transcription factor genes involved in nitrogen mechanism such as LBD37-like genes and stress-response genes such as *DREB1A, IRO2*, and *NAC5* (Kan et al., 2015; Muthuramalingam et al., 2020).

Carbohydrates were differentially accumulated in *Arabidopsis* roots and leaves after treatment with SWE. We found that, for example, glucono-1,5-lactone and glucaro-1,5-lactone were strongly increased in abundance while ß-D-xylose was significantly decreased in treated leaf and root samples. Glucono-1,5-lactone is a carbohydrate that takes part in the pentose phosphate pathway. High concentrations of glucono-1,5-lactone and gluconic acid were observed in *Poa crymophila*, a species that is greatly adaptable to long-term high and low temperatures and drought (Wang et al., 2021). Glucaro-1,5-lactone can be converted to glucaro-1,4 lactone, which has been suggested to enable protection of lipids and proteins against oxidative damage, such as that due to H_2_O_2_ (Olas et al., 2007). ß-D-xylose is a major component of hemicellulose and is required for the structure of cell walls, which may contribute to the prevention of cell breakdown due to dehydration (Zhang et al., 2021). Broadly, a change in xylose has been observed in various species under different stress conditions, indicating the important roles of xylose in tolerance to abiotic stress (Lima et al., 2013; Le Gall et al., 2015). For example, Viana et al. (2022) showed that in canola (*Brassica napus L.*), there was a 190% increase in xylose content in roots under salt stress, while there was a 36% decrease in accumulation of this compound in shoots under drought stress.

It is worth noting that the relative abundance of malonic acid, among other organic acids, was found to be highly accumulated in the leaves but not in the roots at all investigated time points. In contrast, malonic acid was recorded to be the most abundant organic acid in the roots of chickpea (*Cicer arietinum* L.). Li and Copeland (2000) suggested that malonic acid was a defensive compound enabling drought stress tolerance. In addition, in *Digitalis lanata*, Igamberdiev and Eprintsev (2016) and Groeneveld et al. (1992) have shown that malonic acid can be metabolized from malonyl-CoA to become a precursor for neutral lipids and cardenolide defence metabolites.

We have also found that the levels of the vitamin ascorbic acid and D-glucuronic acid (an intermediate in ascorbic acid biosynthesis) were rapidly enhanced in the leaves harvested immediately after applying the second SWE treatment (day 0). Ascorbic acid is a non-enzymatic antioxidant that can potentially reduce oxidative damage, contribute to the mediation of fundamental mechanisms such as photosynthesis, biosynthesis of hormones or other antioxidants during stress or in non-stress environments (Akram et al., 2017; Garcia-Garcia et al., 2020). An increased concentration of ascorbic acid was also found after treatment with other biostimulants such as a silicon-based biostimulant (Chele et al., 2021) and hexanoic acid (Camanes et al., 2015).

Similar to vitamins, hormones play major roles in plant growth and adaptations to environmental changes. In our study, differential changes in the relative content of salicylic acid (SA), indole-3-acetic acid (IAA) and its derivatives (IAA-glucose and oxo-IAA) were identified in the *Arabidopsis* roots following SWE treatment (**Figure 8**). SA is a phenolic compound that is involved in many plant processes such as seed germination, photosynthesis, flowering and senescence (Rivas-San Vicente and Plasencia, 2011). The SA signaling pathway is involved in activating systemic acquired resistance (SAR) under adverse conditions, which is the long-distance transport of defence signals to enhance responses against secondary attack (Zeier, 2021). Previous studies have indicated that increases in SA content were induced by seaweed-derived bioactive compounds in various species such as tomato (El Modafar et al., 2012), *Arabidopsis* (Zhang et al., 2019) or blue gum (*Eucalyptus globulus*) (Shukla et al., 2016). SA-mediated regulation was dependent on the interaction and cross-talk with other stress-induced signaling molecules such as pipecolic acid, jasmonic acid and ethylene; or other plant growth-related phytohormones such as auxin or abscisic acid (Navarro et al., 2008; Shields et al., 2022; Singh et al., 2022).In addition to SA, IAA is the main type of auxin in plants that contributes to fundamental physiological and biochemical processes (Zhao, 2010). In our study, a high abundance of IAA-glucose, an inactive form of IAA (Korasick et al., 2013) was observed at day 0 while the active form, IAA and its degraded product (oxIAA) were reduced. Low concentrations of free IAA and its intermediates in plants were suggested to be associated with plant growth enhancement, especially root growth promotion (Thimann, 1937; Pilet and Saugy, 1987; Ivanchenko et al., 2010). Therefore, the reduced accumulation of IAA and its derivatives in the current study may indicate that treatment with SWE leads to plant growth promotion.

### 4.2 Seaweed extracts triggered changes in secondary metabolites for defence and signaling systems

We have shown that several compounds related to phenylpropanoid metabolism were significantly altered by the treatment with SWE in both leaf and root samples. There was a number of compounds that were increased at a specific time following treatment with SWE such as phenylglyoxylic acid in the leaves and 3-methoxyphenylacetic acid in the roots. Accumulation of phenylglyoxylic acid was recorded in *Ricinus communis* cotyledons during salt stress (Wang et al., 2021), while 3-methoxyphenylacetic acid was found accumulated in canola seeds (*Brassica napus* L.) (Misra and Yildiz, 2016). Increased accumulation of phenylpropanoid metabolites has been found for coumaric acid, and chlorogenic acids in maize (Tinte et al., 2022), and for ferulic acid and caffeic acid in pepper (Ertani et al., 2014) that were treated with plant-based or seaweed-based biostimulants *via* root or foliar applications. Other phenylpropanoid compounds identified here, such as vanilloylglycine, N-feruloylglycine, and 5-hydroxyferulic acid were decreased in the SWE-treated plants as compared to the control samples harvested at all sampling times. These three compounds belong to the sub-class hydroxycinnamic acids (HCAs), which is broadly defined as a group of compounds derived from cinnamic acid (El-Seedi et al., 2012). HCAs play an essential role in plant immunity by enhancing antimicrobial effects, strengthening cell walls, and mediating stomatal activity (Liu et al., 2022), which contribute to protecting plants against biotic and abiotic stress (Macoy et al., 2015). Reduced accumulations of phenylpropanoid compounds in our study compared with other studies may be a result of the dependence of their activity on the bioactive components, which are often species specific and even showed differences between extracts derived from the same seaweed species (De Saeger et al., 2019; Ali et al., 2021; Shukla et al., 2021).

We also found significantly increased accumulation following SWE treatment of three glucosinolate intermediates (3-(5’-methylthio) pentylmalic acid, 2-(6’-methylthio) hexylmalic acid, and 3-(7’-methylthio) heptylmalic acid), which contribute to the main chain-elongation process of glucosinolate biosynthesis from methionine. Glucosinolates (GSLs) is a class of defence metabolites, mostly found in *Brassicaceae* species including the model plant *Arabidopsis thaliana* (Martinez-Ballesta et al., 2013). Many studies have demonstrated that the accumulations and composition of GSLs were altered significantly during activation of defence, protecting plants against pathogens and abiotic stresses (Guo et al., 2013; Mitreiter and Gigolashvili, 2021). For example, under drought stress, enhanced accumulation of GSLs in the cytosol of *Arabidopsis* leaves may reduce inward K^+^ channels in the guard cells, resulting in stomatal closure to reduce water loss (Martinez-Ballesta et al., 2013). In addition, in the current study, acrylic acid (a member of ally-GSLs) and its derivative, 3-indoleacrylic acid, were observed with higher relative abundance in SWE-treated roots. Katz et al. (2020) reported that exogenous application of acrylic acid inhibited *Arabidopsis* root growth by altering the cell cycle, indicating the complex functions of GSLs in plant growth and defence.

### 4.3 Metabolic changes associated with seaweed extract-induced priming and systemic responses

Systemic acquired resistance (SAR) is a mechanism activated after the localized exposure to microbial pathogens to provide long-lasting and broad-spectrum resistance to plants (Zeier, 2021). In our study, many metabolites such as malonic acid and three glucosinolate intermediates, which are known to be defence-related metabolites (Igamberdiev and Eprintsev, 2016; Mitreiter and Gigolashvili, 2021), showed high accumulation in the leaves compared to that in the roots and an accumulation that was present for at least 5 days after the SWE treatment. We provide evidence that the seaweed extract, which was initially applied to the roots, induced root to shoot signalling that eventually triggered changes in the leaf metabolic profiles. It is well-established that roots communicate with leaves *via* a wide range of mobile signals (Li et al., 2021), including both chemical and physical signals such as ROS, Ca^2+^, phytohormones and hydraulic and electrical signals which are increased in roots during stress. Here, the SWE-induced root to shoot signalling, therefore, is likely to enhance SAR in *Arabidopsis* once the plants are exposed to a pathogen. Indeed, we previously demonstrated (Islam et al., 2021) that during pathogen attack there was increased production of H_2_O_2_ and up regulation of ROS-related genes such as *RBOHD*, *GSTF8, SAG21*, and *TPX2* in *A. thaliana* and *S. lycopersicum* roots treated with SWE. The mechanism was linked directly with SWE-induced priming, a phenomenon that plants react more rapidly and vigorously to biotic or abiotic stress, through significantly enhanced expression of SAR and priming-related genes.

**Figure 9:**
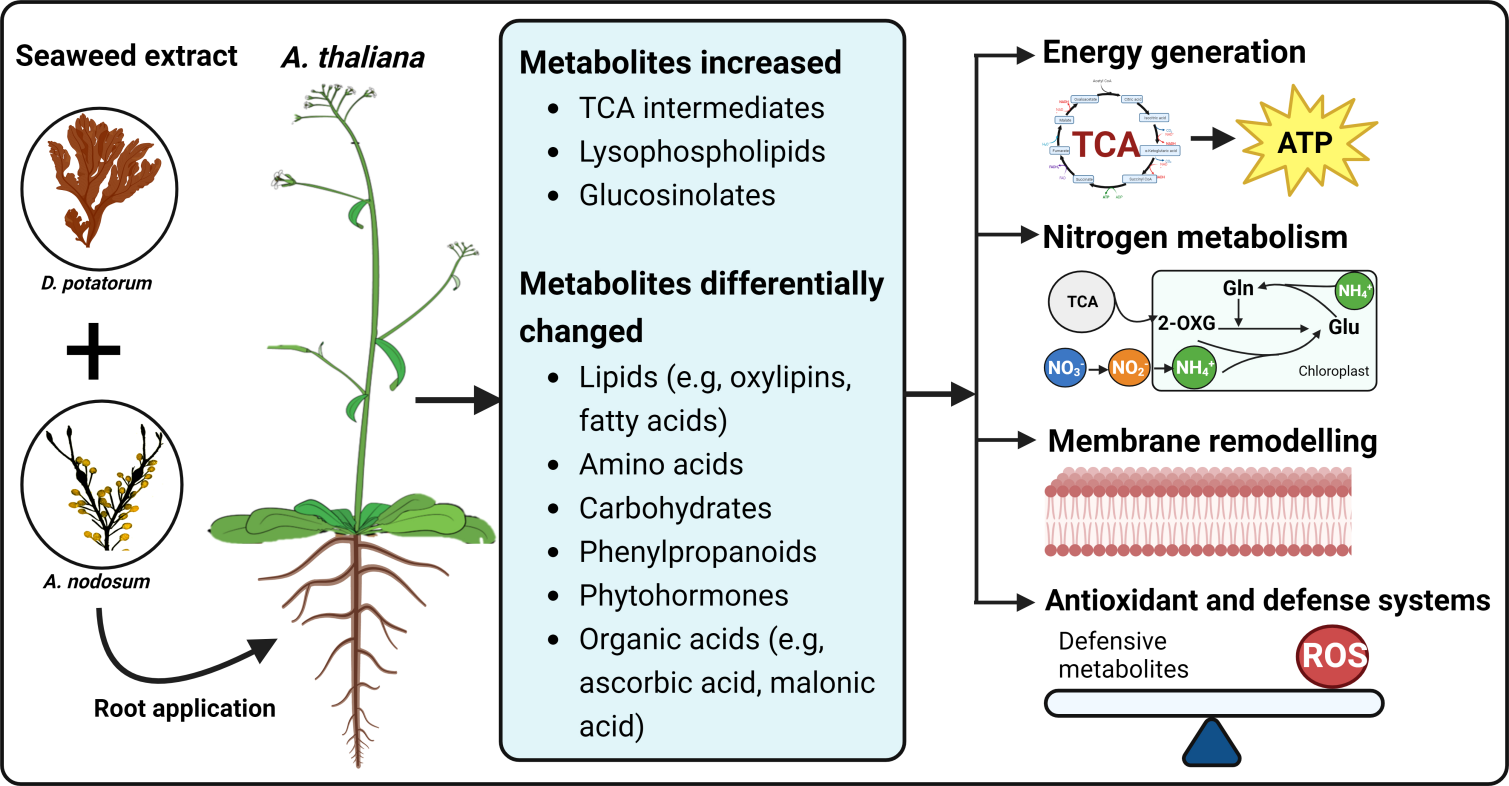
A summary of changes in *Arabidopsis* metabolic profiles induced by seaweed extracts. The SWE, derived from *D. potatorum* and *A. nodosum* were twice applied to *Arabidopsis* roots and metabolic changes were observed after 0, 3, and 5 days after the second treatment. We demonstrated that SWE triggered changes in various important metabolite groups such as lipids, amino acids, carbohydrates and phytohormones. Significant accumulations of essential growth and defence-related metabolites such as TCA cycle, lysophospholipids and glucosinolates were also detected in both SWE-treated leaves and roots. All these metabolic changes may contribute to enhancing energy production, carbon and nitrogen metabolism, remodeling of cell membranes and improving antioxidant and defence systems. The figure was generated using BioRender.

In summary, treatment with SWE clearly triggered a metabolic reprogramming up to 5 days after SWE treatment in both leaf and root tissues. A summary of changes in metabolic profiles of SWE-treated plants is presented in **Figure 9**. Enhanced carbon and nitrogen metabolism, shown by strong accumulation of TCA cycle and N-containing (such as glutamine, glutamic acid, and its derivatives) metabolites, would provide more energy and precursors for fundamental biological and signalling pathways. Changes in lipid and secondary metabolite profiles, including those of phenylpropanoids, glucosinolates, and other organic acids may strengthen defence and antioxidant systems. In our previous studies at the transcriptomic level, we showed enhanced priming and systemic responses in *Arabidopsis*, (Islam et al., 2020; Islam et al., 2021) that we have now aligned to specific changes at the metabolomic level in leaves and roots.

## 5 Conclusion

In conclusion, the current study provides fundamental findings that describe the mechanism for the action of a seaweed extract-based biostimulant on *Arabidopsis*. Untargeted metabolomic analysis of both leaves and roots revealed accumulation of metabolites related to plant growth and priming were induced following application of seaweed extract. We also have shown that metabolic reprogramming has involved the increased accumulation of TCA metabolites and differential changes in the levels of lipids, amino acids, carbohydrates, phytohormones and secondary metabolites such as phenylpropanoids and glucosinolates. Many of these compounds participate in carbon and nitrogen metabolism, which are directly involved in plant growth and development. Importantly, the research indicates the mechanisms induced by seaweed extracts that may benefit plant growth and tolerances to abiotic and biotic stresses. These mechanisms involve (1) a diverse cascade of cellular, gene expression and metabolic responses related to plant growth and stress tolerance, and (2) those that are systemic and enable propagation of plant priming responses. Knowledge generated from this research will provide a foundation to explore further the beneficial effects and applications of seaweed extracts or biostimulants. These investigations may contain targeted identification of key functional compounds and regulation networks. In-depth knowledge derived from biostimulant-related research will contribute to developing an alternative source of fertilizers for more productive and sustainable agriculture.

## Data availability statement

The original contributions presented in the study are included in the article/**Supplementary Material**. Further inquiries can be directed to the corresponding author.

## Author contributions

TT, DCah, and TA conceptualized and designed the project. TT performed the laboratory work, analysed the metabolomic data, wrote and revised the manuscript. MI contributed to growing plants and sample collection. DCal contributed to LC-MS experiments and data processing. DCah, MI, YW, DCal and TA contributed to the manuscript draft. DCah supervised and provided a final approval of the manuscript’s content. All authors revised and approved the final version to be published and agreed with all aspects of the work. All authors contributed to the article and approved the submitted version.
