# Supplementary material for: Comparative metabolomic profiling of Arabidopsis thaliana roots and leaves reveals complex response mechanisms induced by a seaweed extract
Source: Front Plant Sci. 2023 Mar 9;14:1114172. doi: 10.3389/fpls.2023.1114172 (PMC10035662; doi:10.3389/fpls.2023.1114172)
Supplement: Supplementary file 1 [file DataSheet_1.docx]

**Supplementary information**

**Supplementary Figure S1.** UHPLC-MS sample extraction and sequence of measurement.

**Supplementary Figure S2.** Compound Discoverer 3.3.1.111 workflow for the untargeted UHPLC-MS analysis.

**Supplementary Figure S3.** PCA plot showing clustering of quality control (QC) samples.

**Supplementary Figure S4.** Cross validation (A) and permutation tests (B) for the OPLS-DA model.

**Supplementary Table S1.** Detailed settings of the untargeted workflow used for metabolomic data processing.

**Supplementary Table S2.** Metabolites that significantly changed in abundance (p-value < 0.05 and log2FC > 0.6 or < -0.6) was confirmed by using the predicted composition and/or mzCloud matching in combination with FISh scoring and literature.

**Supplementary Table S3.** Reference sources for 89 compounds that were significantly changed in leaves and roots following the application of SWE.

**Supplementary Table S4.** Metabolites that were significantly changed in accumulation in the roots and showed changes or no changes in accumulation in the leaves.

**Supplementary Table S5.** Metabolites that were significantly changed in accumulation in the leaves and showed changes or no changes in accumulation in the roots.

**Supplementary Table S6.** Common metabolites that were significantly changed in accumulation in the roots across all three time points.

**Supplementary Table S7**. Common metabolites that were significantly changed in accumulation in the leaves across all three time points.


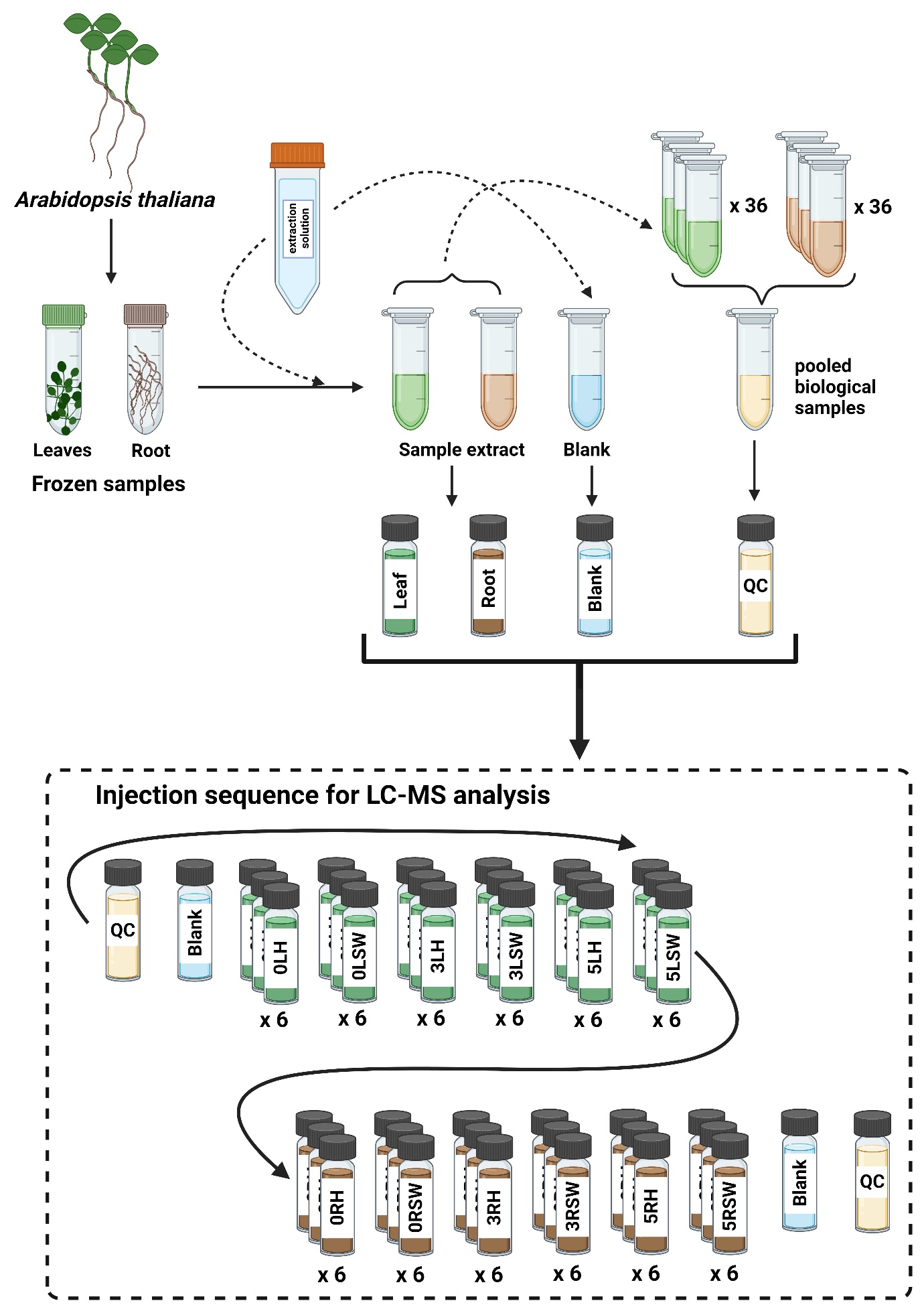


**Supplementary Figure S1. UHPLC-MS sample extraction and sequence of measurement.** Roots and leaves were separated from non-treated and SWE-treated *Arabidopsis* plants and stored at -80°C. Frozen root and leaf samples were then extracted to prepare samples for UHPLC-MS measurement. Two blank samples containing only extraction solution were used for the detection and identification of background compounds, which were subsequently removed. The two pooled biological quality control samples were created by mixing equally all individual samples together, and it was analyzed at the beginning and at the end of the UHPLC-MS run. The injection order was not randomized, and the details for the injection sequence are presented. The numbers '0', '3', and '5' represent 'day 0', 'day 3', and 'day 5' samples, respectively. The letters' L' and 'R' represent leaves and root samples, respectively. The letters 'H' and 'SW' represent control and seaweed extract treated samples, respectively. The figure was generated using BioRender.
