# Supplementary material for: Comparative metabolomic profiling of Arabidopsis thaliana roots and leaves reveals complex response mechanisms induced by a seaweed extract
Source: Front Plant Sci. 2023 Mar 9;14:1114172. doi: 10.3389/fpls.2023.1114172 (PMC10035662; doi:10.3389/fpls.2023.1114172)
Supplement: Supplementary file 2 [file DataSheet_2.docx]

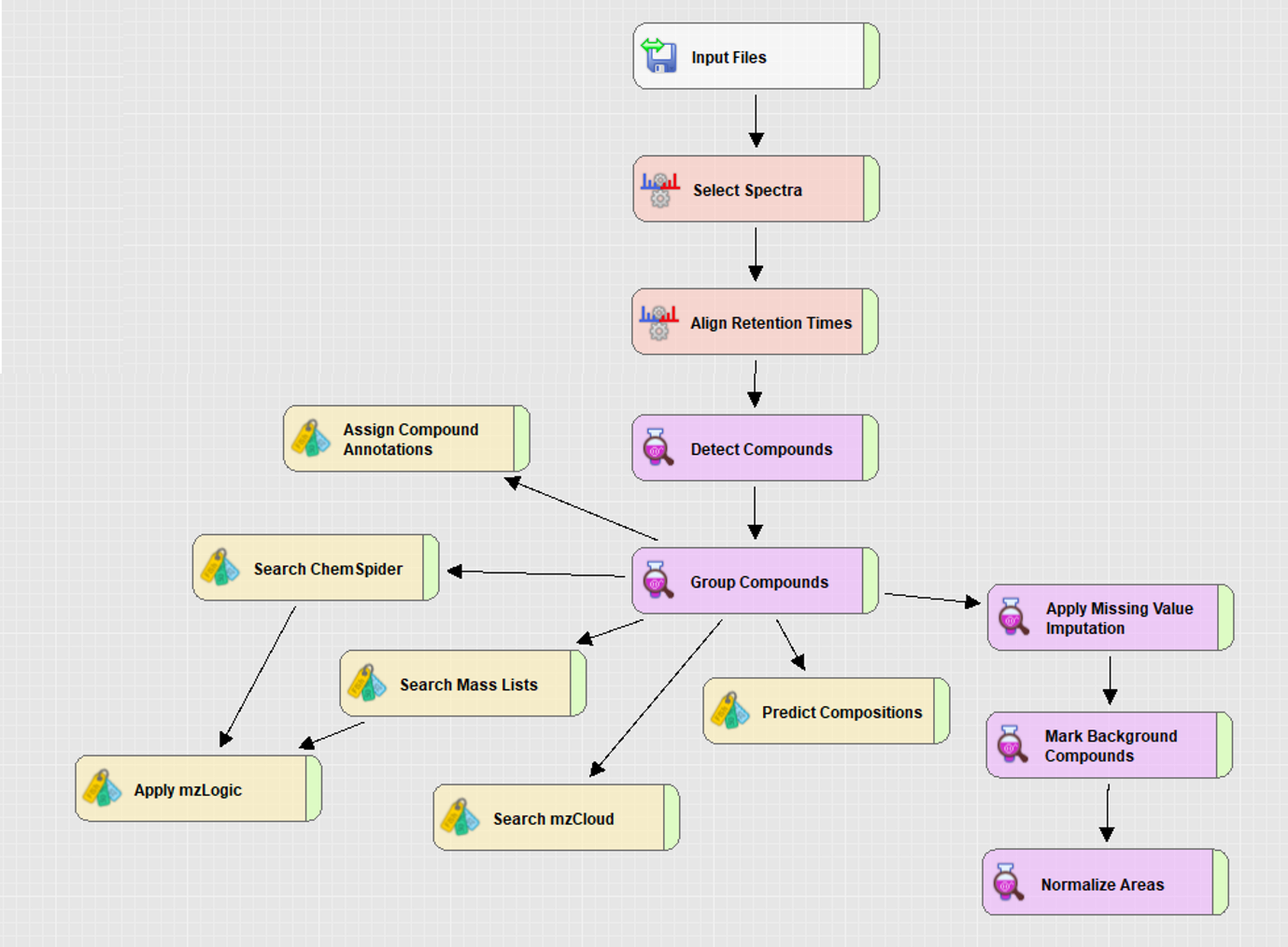


**Supplementary Figure S2.** Compound Discoverer 3.3.1.111 workflow for the untargeted UHPLC-MS analysis. The untargeted workflow named “Untargeted Metabolomics with Statistics Detect Unknowns with ID using Online Databases and mzLogic” (Thermo Fisher Scientific, USA) with some modifications was applied.
