# Supplementary material for: Comparative metabolomic profiling of Arabidopsis thaliana roots and leaves reveals complex response mechanisms induced by a seaweed extract
Source: Front Plant Sci. 2023 Mar 9;14:1114172. doi: 10.3389/fpls.2023.1114172 (PMC10035662; doi:10.3389/fpls.2023.1114172)
Supplement: Supplementary file 3 [file DataSheet_3.docx]

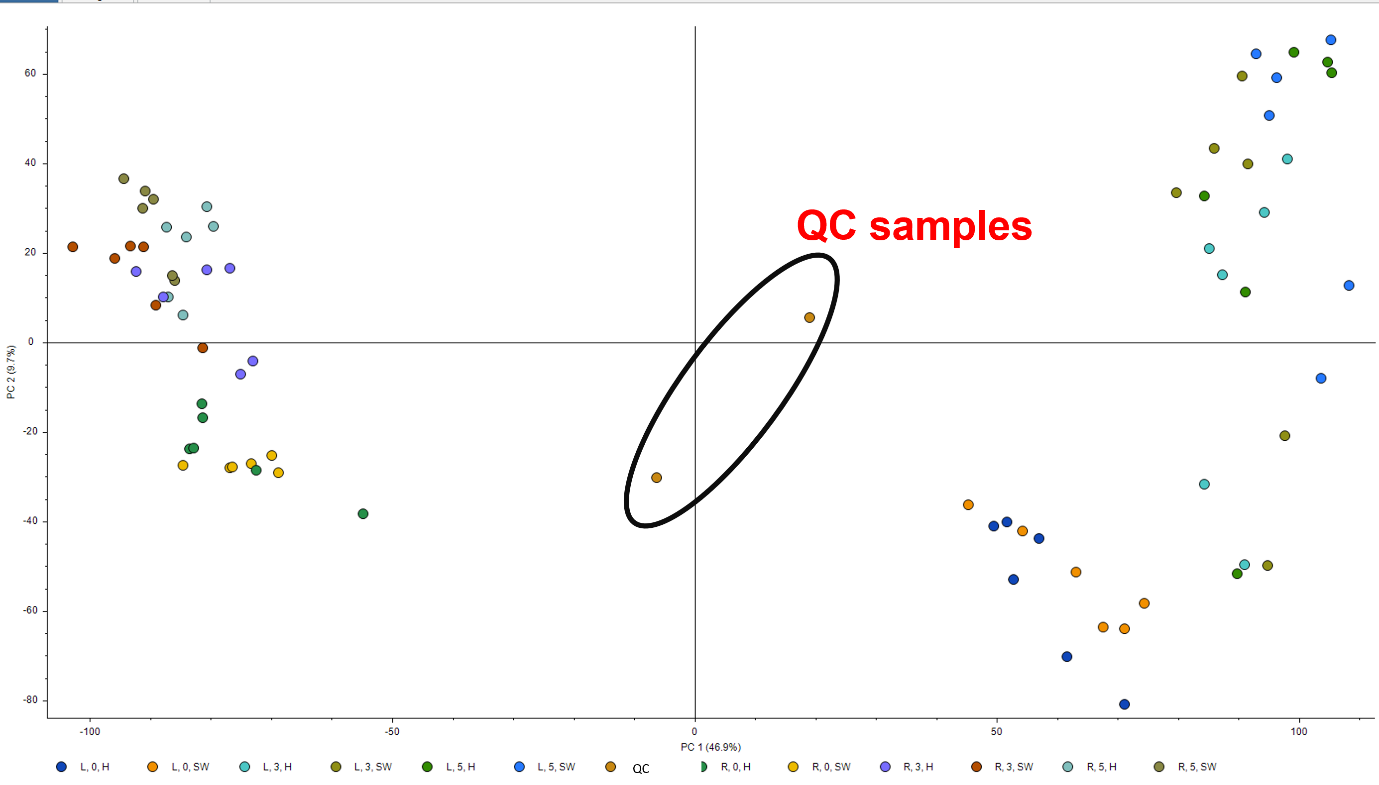
 **Supplementary Figure S3. PCA plot showing clustering of quality control (QC) samples.** The letters ‘L’ and ‘R’ represents leaf and root samples, respectively. The numbers ‘0’, ‘3’, and ‘5’ represent day 0, day 3 and day 5 samples, respectively. The letters ‘H’ and ‘SW’ represent control and SWE-treated samples, respectively.
