# Supplementary material for: Comparative metabolomic profiling of Arabidopsis thaliana roots and leaves reveals complex response mechanisms induced by a seaweed extract
Source: Front Plant Sci. 2023 Mar 9;14:1114172. doi: 10.3389/fpls.2023.1114172 (PMC10035662; doi:10.3389/fpls.2023.1114172)
Supplement: Supplementary file 4 [file DataSheet_4.docx]

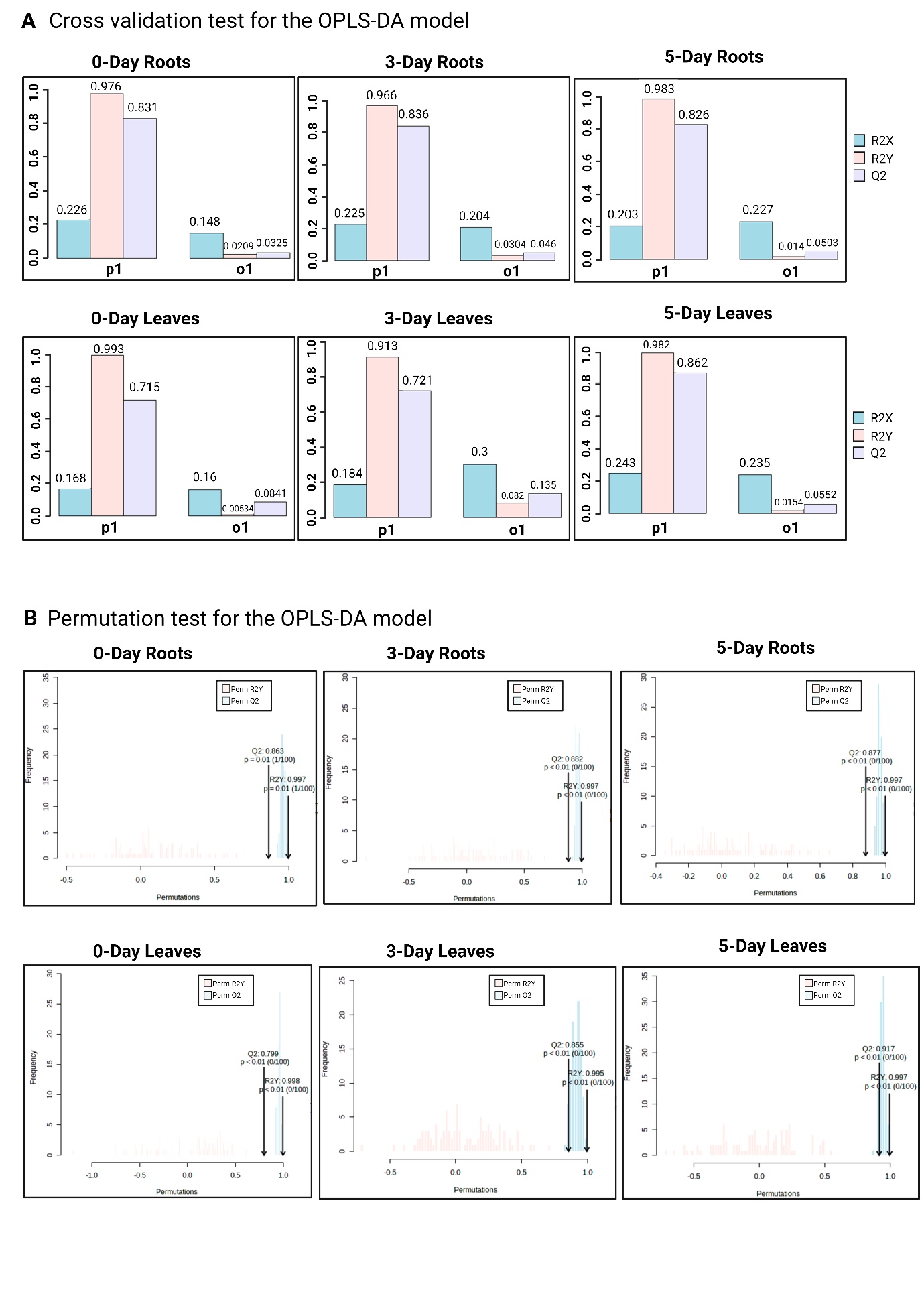
**Supplementary Figure S4.** **Cross validation (A) and permutation tests (B) for the OPLS-DA model.** The results indicated that the discrimination between the two compared groups of the model is statistically significant and reliable.
