# Supplementary material for: Comparative metabolomic profiling of Arabidopsis thaliana roots and leaves reveals complex response mechanisms induced by a seaweed extract
Source: Front Plant Sci. 2023 Mar 9;14:1114172. doi: 10.3389/fpls.2023.1114172 (PMC10035662; doi:10.3389/fpls.2023.1114172)
Supplement: Supplementary file 7 [file Table_3.docx]

**Supplementary Table S3.** Reference sources for 89 compounds that were significantly changed in leaves and roots following the application of SWE.

| **Metabolites** | **Reference** |
| --- | --- |
| **1. Lipids and related compounds** | |
| **1.1 fatty acids and related compounds** | |
| 1-Linoleoyl glycerol | Aftab et al. (2020) |
| Hexadecanoic acid | Hu et al. (2018) |
| Octanoic acid | Yasuno & Wada (1998), Ramadan et al. (2022) |
| Corchorifatty acid F | Li et al. (2020) |
| Itaconic acid | Wang et al. (2021) |
| Crotonic acid | Jasicka-Misiak et al. (2005) |
| **1.2 α-linolenic acid and related compounds** | |
| 12-OPDA | Huang et al. (2017) |
| 13-HPOT | Ruan et al. (2019) |
| Traumatic Acid | Pietryczuk et al. (2013) |
| OPC-8:0 related | Ruan et al. (2019) |
| JA-Ile | Huang et al. (2017), Browse and Howe (2008) |
| 12-Hydroxy JA | Wasternack (2007) |
| Cucurbic acid | Browse and Howe (2008) |
| Jasmonic acid | Ghorbel et al. (2021) |
| **1.3 linoleic acid and related compounds** | |
| 9(S)-HPODE | Singh et al. (2022) |
| 9,12,13-TriHOME | Offor et al. (2022) |
| 9-KODE | Montillet et al. (2013) |
| **1.4 Lysophospholipids** | |
| LysoPE(18:2/0:0) | Rocchetti et al. (2020) |
| LysoPC(18:2/0:0) | Cho et al. (2012), Galati et al. (2019) |
| LysoPE(18:0/0:0) | Volz et al. (2021) |
| LysoPE(16:1/0:0) | Volz et al. (2021) |
| **1.5 Other lipids** | |
| Linoleoyl ethanolamide | Gachet et al. (2017) |
| α-Linolenoyl ethanolamide | Gachet et al. (2017) |
| **2. Amino acids and related compounds** | |
| D-Glutamine | Miflin & Habash (2002) |
| γ-L-Glutamyl-L-glutamic acid | Yang et al. (2020) |
| N-Acetyl-D-alloisoleucine | Shen et al. (2021) |
| L-Glutamic acid | Forde and Lea (2007), Qiu et al. (2019) |
| L-Serine | Zeier (2013) |
| L-Asparagine | Han et al. (2021) |
| H-(γ-Glu)_2_-Glu-OH | Yang et al. (2020) |
| N-Phenyl-L-glutamine | Miflin & Habash (2002) |
| L-Arogenic acid | Schenck and Maeda (2018) |
| N-Acetylvaline | Xiao et al. (2020) |
| Proline | Khan et al. (2019), Goni et al. (2018) |
| L-Pyroglutamic acid | Mwamba et al. (2020) |
| **3. Carbonhydrates and related compounds** | |
| Glyceric acid | Khan et al. (2019) |
| D-Glucono-1,5-lactone | Kurotani et al. (2022) |
| D-Glucose 6-phosphate | Hu et al. (2019) |
| D-Glucuronate | Viana et al. (2022) |
| beta-D-Xylose | Zhang et al. (2021) |
| Zizybeoside II | Fuel et al. (2021), Li et al. (2014) |
| D-glucaro-1,5-lactone | Mwamba et al. (2020) |
| 4-laurylsucrose | Fan et al. (2016) |
| Glucoheptonic Acid | Liu et al. (2015) |
| **4. TCA-cycle and related compounds** | |
| Citric acid | Gao et al. (2020) |
| Fumaric acid | Schwachtje et al. (2018) |
| Succinic semialdehyde | Bouche et al. (2003) |
| DL-Malic acid | Schwachtje et al. (2018) |
| 2-Oxoglutaric acid | Araujo et al. (2014) |
| trans-Aconitic acid | Igamberdiev and Eprintsev (2016) |
| Oxalosuccinic acid | Guo et al. (2022) |
| **5. Phytohormones and related compounds** | |
| IAA-glucose | Michalczuk and Bandurski (1982), Korasick et al. (2013) |
| OxIAA | Lephatsi et al. (2022) |
| Indole-3-acetic acid | Lephatsi et al. (2022), Korasick et al. (2013) |
| Salicylic acid | Shields et al. (2022) |
| **6. Nucleotides and related compounds** | |
| Cytosine | Biswas et al. (2018) |
| 5,6-Dihydrothymidine | Griffiths et al. (1998) |
| Adenine | Gao et al. (2020) |
| **7. Vitamins** | |
| Ascorbic acid | Akram et al. (2017) |
| **8. Phenylpropanoids and related compounds** | |
| N-feruloylglycine | Tharanathan (2002), Valette et al. (2020) |
| Vanilloylglycine | Kumar and Pruthi (2014) |
| 5-Hydroxyferulic acid | Misra (2016) |
| p-coumaroylagmatine | Muroi et al. (2009) |
| 1-O-(4-coumaroyl)-beta-D-glucose | Pei et al. (2016) |
| 3-Methoxyphenylacetic acid | Misra (2016) |
| Grandisin | Marques et al. (2022) |
| **9. Glucosinolates and related compounds** | |
| 2-(6'-methylthio)hexylmalic acid | Kitainda and Jez (2021), Textor et al. (2004) |
| 3-(5'-methylthio)pentylmalic acid | Kitainda and Jez (2021), Textor et al. (2004) |
| 3-(7'-methylthio)heptylmalic acid | Kitainda and Jez (2021), Textor et al. (2004) |
| Acrylic acid | Katz et al. (2020) |
| 3-Indoleacrylic acid | Kumar et al. (2021) |
| **10. Organic acids** | |
| 3-Oxododecanoic acid | Zandona et al. (2020) |
| Levulinic acid | Akram and Ashraf (2013), Wu et al. (2018) |
| 3-Hydroxybutyric acid | Li et al. (2017) |
| Malonic acid | Li and Copeland (2000) |
| Oxopent-4-enoate | Muto et al. (2013) |
| **11. Flavonoids** | |
| Luteolin | Du et al. (2010) |
| kaempferol 7-O-ß-D-glucopyranoside | Lee et al. (2017) |
| Licoisoflavone A | War et al. (2012) |
| **12. Benzenoids** | |
| 2-anisaldehyde | Szelenyi et al. (2020) |
| Phenylglyoxylic acid | Wang et al. (2021) |
| 6-Decylubiquinol | (Gao et al., 2020) |
| **13. Alkaloids** | |
| Buchananine | Sidiq et al. (2021) |
| **14. Others** | |
| 3-O-(α-L-oleandrosyl)oleandolide | Martinez et al. (2022) |
| N-Benzyloxycarbonylglycine | Yang et al. (2020) |
| Encecalin | Hernandez-Altamirano et al. (2020) |
| Methyl vinyl ketone | Tani et al. (2020) |
| Picolinic acid | Pan et al. (2018), Aucique-Perez et al. (2019) |
| Olivetol | Sirikantaramas and Taura (2017) |

**References**

Aftab, A., Yousaf, Z., Aftab, Z. E., Younas, A., Riaz, N., Rashid, M., Shamsheer, H. B., Razzaq, Z., & Javaid, A. (2020). Pharmacological screening and GC-MS analysis of vegetative/reproductive parts of Nigella sativa L. *Pakistan journal of pharmaceutical sciences*, *33*(5), 2103–2111.

Ben J. Miflin, Dimah Z. Habash, The role of glutamine synthetase and glutamate dehydrogenase in nitrogen assimilation and possibilities for improvement in the nitrogen utilization of crops, Journal of Experimental Botany, Volume 53, Issue 370, 15 April 2002, Pages 979–987, <https://doi.org/10.1093/jexbot/53.370.979>

Bouche, N., Fait, A., Bouchez, D., Moller, S. G., & Fromm, H. (2003). *Mitochondrial succinic-semialdehyde dehydrogenase of the  -aminobutyrate shunt is required to restrict levels of reactive oxygen intermediates in plants. Proceedings of the National Academy of Sciences, 100(11), 6843–6848.* doi:10.1073/pnas.1037532100

Michalczuk, L., & Bandurski, R. S. (1982). Enzymic synthesis of 1-O-indol-3-ylacetyl-beta-D-glucose and indol-3-ylacetyl-myo-inositol. *The Biochemical journal*, *207*(2), 273–281. <https://doi.org/10.1042/bj2070273>

Griffiths, H. R., Mistry, P., Herbert, K. E., & Lunec, J. (1998). Molecular and cellular effects of ultraviolet light-induced genotoxicity. *Critical reviews in clinical laboratory sciences*, *35*(3), 189–237. <https://doi.org/10.1080/10408369891234192>

Tharanathan R. N. (2002). Food-derived carbohydrates--structural complexity and functional diversity. *Critical reviews in biotechnology*, *22*(1), 65–84. <https://doi.org/10.1080/07388550290789469>

Biswapriya Biswavas Misra | Fatih Yildiz (Reviewing Editor) (2016) Cataloging the *Brassica napus* seed metabolome, Cogent Food & Agriculture, 2:1, DOI: [10.1080/23311932.2016.1254420](https://doi.org/10.1080/23311932.2016.1254420)

Li, J., & Copeland, L. (2000). Role of malonate in chickpeas. *Phytochemistry*, *54*(6), 585–589. https://doi.org/10.1016/s0031-9422(00)00162-x

Akram, N.A., and Ashraf, M. (2013). Regulation in Plant Stress Tolerance by a Potential Plant Growth Regulator, 5-Aminolevulinic Acid. *Journal of Plant Growth Regulation* 32(3)**,** 663-679. doi: 10.1007/s00344-013-9325-9.

Akram, N.A., Shafiq, F., and Ashraf, M. (2017). Ascorbic Acid-A Potential Oxidant Scavenger and Its Role in Plant Development and Abiotic Stress Tolerance. *Front Plant Sci* 8**,** 613. doi: 10.3389/fpls.2017.00613.

Araujo, W.L., Martins, A.O., Fernie, A.R., and Tohge, T. (2014). 2-Oxoglutarate: linking TCA cycle function with amino acid, glucosinolate, flavonoid, alkaloid, and gibberellin biosynthesis. *Front Plant Sci* 5**,** 552. doi: 10.3389/fpls.2014.00552.

Aucique-Perez, C.E., Resende, R.S., Neto, L.B.C., Dornelas, F., DaMatta, F.M., and Rodrigues, F.A. (2019). Picolinic acid spray stimulates the antioxidative metabolism and minimizes impairments on photosynthesis on wheat leaves infected by Pyricularia oryzae. *Physiol Plant* 167(4)**,** 628-644. doi: 10.1111/ppl.12917.

Biswas, S., Biswas, A.K., and De, B. (2018). Metabolomics analysis of Cajanus cajan L. seedlings unravelled amelioration of stress induced responses to salinity after halopriming of seeds. *Plant Signal Behav* 13(7)**,** e1489670. doi: 10.1080/15592324.2018.1489670.

Browse, J., and Howe, G.A. (2008). New weapons and a rapid response against insect attack. *Plant Physiol* 146(3)**,** 832-838. doi: 10.1104/pp.107.115683.

Cho, K., Kim, Y., Wi, S.J., Seo, J.B., Kwon, J., Chung, J.H., et al. (2012). Nontargeted metabolite profiling in compatible pathogen-inoculated tobacco (Nicotiana tabacum L. cv. Wisconsin 38) using UPLC-Q-TOF/MS. *J Agric Food Chem* 60(44)**,** 11015-11028. doi: 10.1021/jf303702j.

Du, Y., Chu, H., Wang, M., Chu, I.K., and Lo, C. (2010). Identification of flavone phytoalexins and a pathogen-inducible flavone synthase II gene (SbFNSII) in sorghum. *J Exp Bot* 61(4)**,** 983-994. doi: 10.1093/jxb/erp364.

Fan, P., Miller, A.M., Schilmiller, A.L., Liu, X., Ofner, I., Jones, A.D., et al. (2016). In vitro reconstruction and analysis of evolutionary variation of the tomato acylsucrose metabolic network. *Proc Natl Acad Sci U S A* 113(2)**,** E239-248. doi: 10.1073/pnas.1517930113.

Forde, B.G., and Lea, P.J. (2007). Glutamate in plants: metabolism, regulation, and signalling. *J Exp Bot* 58(9)**,** 2339-2358. doi: 10.1093/jxb/erm121.

Fuel, M., Mesas, C., Martinez, R., Ortiz, R., Quinonero, F., Prados, J., et al. (2021). Antioxidant and antiproliferative potential of ethanolic extracts from Moringa oleifera, Tropaeolum tuberosum and Annona cherimola in colorrectal cancer cells. *Biomed Pharmacother* 143**,** 112248. doi: 10.1016/j.biopha.2021.112248.

Gachet, M.S., Schubert, A., Calarco, S., Boccard, J., and Gertsch, J. (2017). Targeted metabolomics shows plasticity in the evolution of signaling lipids and uncovers old and new endocannabinoids in the plant kingdom. *Sci Rep* 7**,** 41177. doi: 10.1038/srep41177.

Galati, G., Gandin, A., Jolivet, Y., Larbat, R., and Hehn, A. (2019). Untargeted Metabolomics Approach Reveals Diverse Responses of Pastinaca Sativa to Ozone and Wounding Stresses. *Metabolites* 9(7). doi: 10.3390/metabo9070153.

Gao, H., Zhou, Q., Yang, L., Zhang, K., Ma, Y., and Xu, Z.Q. (2020). Metabolomics analysis identifies metabolites associated with systemic acquired resistance in Arabidopsis. *PeerJ* 8**,** e10047. doi: 10.7717/peerj.10047.

Ghorbel, M., Brini, F., Sharma, A., and Landi, M. (2021). Role of jasmonic acid in plants: the molecular point of view. *Plant Cell Rep* 40(8)**,** 1471-1494. doi: 10.1007/s00299-021-02687-4.

Goni, O., Quille, P., and O'Connell, S. (2018). Ascophyllum nodosum extract biostimulants and their role in enhancing tolerance to drought stress in tomato plants. *Plant Physiol Biochem* 126**,** 63-73. doi: 10.1016/j.plaphy.2018.02.024.

Guo, Q., Han, J., Li, C., Hou, X., Zhao, C., Wang, Q., et al. (2022). Defining key metabolic roles in osmotic adjustment and ROS homeostasis in the recretohalophyte Karelinia caspia under salt stress. *Physiol Plant* 174(2)**,** e13663. doi: 10.1111/ppl.13663.

Han, M., Zhang, C., Suglo, P., Sun, S., Wang, M., and Su, T. (2021). l-Aspartate: An Essential Metabolite for Plant Growth and Stress Acclimation. *Molecules* 26(7). doi: 10.3390/molecules26071887.

Hernandez-Altamirano, J.M., Ugidos, I.F., Palazon, J., Bonfill, M., Garcia-Angulo, P., Alvarez, J., et al. (2020). Production of Encecalin in Cell Cultures and Hairy Roots of Helianthella quinquenervis (Hook.) A. Gray. *Molecules* 25(14). doi: 10.3390/molecules25143231.

Hu, L., Bi, A., Hu, Z., Amombo, E., Li, H., and Fu, J. (2018). Antioxidant Metabolism, Photosystem II, and Fatty Acid Composition of Two Tall Fescue Genotypes With Different Heat Tolerance Under High Temperature Stress. *Front Plant Sci* 9**,** 1242. doi: 10.3389/fpls.2018.01242.

Hu, Y., You, J., Li, J., and Wang, C. (2019). Loss of cytosolic glucose-6-phosphate dehydrogenase increases the susceptibility of Arabidopsis thaliana to root-knot nematode infection. *Ann Bot* 123(1)**,** 37-46. doi: 10.1093/aob/mcy124.

Huang, H., Liu, B., Liu, L., and Song, S. (2017). Jasmonate action in plant growth and development. *J Exp Bot* 68(6)**,** 1349-1359. doi: 10.1093/jxb/erw495.

Igamberdiev, A.U., and Eprintsev, A.T. (2016). Organic Acids: The Pools of Fixed Carbon Involved in Redox Regulation and Energy Balance in Higher Plants. *Front Plant Sci* 7**,** 1042. doi: 10.3389/fpls.2016.01042.

Jasicka-Misiak, I., Wieczorek, P.P., and Kafarski, P. (2005). Crotonic acid as a bioactive factor in carrot seeds (Daucus carota L.). *Phytochemistry* 66(12)**,** 1485-1491. doi: 10.1016/j.phytochem.2005.04.005.

Katz, E., Bagchi, R., Jeschke, V., Rasmussen, A.R.M., Hopper, A., Burow, M., et al. (2020). Diverse Allyl Glucosinolate Catabolites Independently Influence Root Growth and Development. *Plant Physiol* 183(3)**,** 1376-1390. doi: 10.1104/pp.20.00170.

Khan, N., Bano, A., Rahman, M.A., Rathinasabapathi, B., and Babar, M.A. (2019). UPLC-HRMS-based untargeted metabolic profiling reveals changes in chickpea (Cicer arietinum) metabolome following long-term drought stress. *Plant Cell Environ* 42(1)**,** 115-132. doi: 10.1111/pce.13195.

Kitainda, V., and Jez, J.M. (2021). Structural Studies of Aliphatic Glucosinolate Chain-Elongation Enzymes. *Antioxidants (Basel)* 10(9). doi: 10.3390/antiox10091500.

Korasick, D.A., Enders, T.A., and Strader, L.C. (2013). Auxin biosynthesis and storage forms. *J Exp Bot* 64(9)**,** 2541-2555. doi: 10.1093/jxb/ert080.

Kumar, N., and Pruthi, V. (2014). Potential applications of ferulic acid from natural sources. *Biotechnol Rep (Amst)* 4**,** 86-93. doi: 10.1016/j.btre.2014.09.002.

Kumar, P., Lee, J.H., and Lee, J. (2021). Diverse roles of microbial indole compounds in eukaryotic systems. *Biol Rev Camb Philos Soc* 96(6)**,** 2522-2545. doi: 10.1111/brv.12765.

Kurotani, K.I., Huang, C., Okayasu, K., Suzuki, T., Ichihashi, Y., Shirasu, K., et al. (2022). Interfamily grafting capacity of petunia. *Hortic Res*. doi: 10.1093/hr/uhab056.

Lee, W.J., Kim, J., Lee, D., Hong, S.-W., and Lee, H. (2017). Arabidopsis UDP-glycosyltransferase 78D1-overexpressing plants accumulate higher levels of kaempferol 3-O-β-d-glucopyranoside than wild-type plants. *Applied Biological Chemistry* 60(6)**,** 647-652. doi: 10.1007/s13765-017-0322-8.

Lephatsi, M., Nephali, L., Meyer, V., Piater, L.A., Buthelezi, N., Dubery, I.A., et al. (2022). Molecular mechanisms associated with microbial biostimulant-mediated growth enhancement, priming and drought stress tolerance in maize plants. *Sci Rep* 12(1)**,** 10450. doi: 10.1038/s41598-022-14570-7.

Li, B., Tao, W., Zheng, C., Shar, P.A., Huang, C., Fu, Y., et al. (2014). Systems pharmacology-based approach for dissecting the addition and subtraction theory of traditional Chinese medicine: An example using Xiao-Chaihu-Decoction and Da-Chaihu-Decoction. *Comput Biol Med* 53**,** 19-29. doi: 10.1016/j.compbiomed.2014.05.007.

Li, M., Guo, R., Jiao, Y., Jin, X., Zhang, H., and Shi, L. (2017). Comparison of Salt Tolerance in Soja Based on Metabolomics of Seedling Roots. *Front Plant Sci* 8**,** 1101. doi: 10.3389/fpls.2017.01101.

Li, P., Xiao, Z., Sun, J., Oyang, X., Xie, X., Li, Z., et al. (2020). Metabolic regulations in lettuce root under combined exposure to perfluorooctanoic acid and perfluorooctane sulfonate in hydroponic media. *Sci Total Environ* 726**,** 138382. doi: 10.1016/j.scitotenv.2020.138382.

Liu, G., Dong, X., Liu, L., Wu, L., Peng, S., and Jiang, C. (2015). Metabolic profiling reveals altered pattern of central metabolism in navel orange plants as a result of boron deficiency. *Physiol Plant* 153(4)**,** 513-524. doi: 10.1111/ppl.12279.

Marques, A.M., da Rocha Queiroz, A.S., Guimaraes, E.F., Mafud, A.C., de Sousa Carvalho, P., Mascarenhas, Y.P., et al. (2022). Piper tectoniifolium Kunth: A New Natural Source of the Bioactive Neolignan (-)-Grandisin. *Molecules* 27(4). doi: 10.3390/molecules27041151.

Martinez, R., Garcia-Beltran, A., Kapravelou, G., Mesas, C., Cabeza, L., Perazzoli, G., et al. (2022). In Vivo Nutritional Assessment of the Microalga Nannochloropsis gaditana and Evaluation of the Antioxidant and Antiproliferative Capacity of Its Functional Extracts. *Mar Drugs* 20(5). doi: 10.3390/md20050318.

Montillet, J.L., Leonhardt, N., Mondy, S., Tranchimand, S., Rumeau, D., Boudsocq, M., et al. (2013). An abscisic acid-independent oxylipin pathway controls stomatal closure and immune defense in Arabidopsis. *PLoS Biol* 11(3)**,** e1001513. doi: 10.1371/journal.pbio.1001513.

Muroi, A., Ishihara, A., Tanaka, C., Ishizuka, A., Takabayashi, J., Miyoshi, H., et al. (2009). Accumulation of hydroxycinnamic acid amides induced by pathogen infection and identification of agmatine coumaroyltransferase in Arabidopsis thaliana. *Planta* 230(3)**,** 517-527. doi: 10.1007/s00425-009-0960-0.

Muto, A., Kotera, M., Tokimatsu, T., Nakagawa, Z., Goto, S., and Kanehisa, M. (2013). Modular architecture of metabolic pathways revealed by conserved sequences of reactions. *J Chem Inf Model* 53(3)**,** 613-622. doi: 10.1021/ci3005379.

Mwamba, T.M., Islam, F., Ali, B., Lwalaba, J.L.W., Gill, R.A., Zhang, F., et al. (2020). Comparative metabolomic responses of low- and high-cadmium accumulating genotypes reveal the cadmium adaptive mechanism in Brassica napus. *Chemosphere* 250**,** 126308. doi: 10.1016/j.chemosphere.2020.126308.

Offor, B.C., Mhlongo, M.I., Steenkamp, P.A., Dubery, I.A., and Piater, L.A. (2022). Untargeted Metabolomics Profiling of Arabidopsis WT, lbr-2-2 and bak1-4 Mutants Following Treatment with Two LPS Chemotypes. *Metabolites* 12(5). doi: 10.3390/metabo12050379.

Pan, L., Meng, C., Wang, J., Ma, X., Fan, X., Yang, Z., et al. (2018). Integrated omics data of two annual ryegrass (Lolium multiflorum L.) genotypes reveals core metabolic processes under drought stress. *BMC Plant Biol* 18(1)**,** 26. doi: 10.1186/s12870-018-1239-z.

Pei, K., Ou, J., Huang, J., and Ou, S. (2016). p-Coumaric acid and its conjugates: dietary sources, pharmacokinetic properties and biological activities. *J Sci Food Agric* 96(9)**,** 2952-2962. doi: 10.1002/jsfa.7578.

Pietryczuk, A., Biziewska, I., Imierska, M., and Czerpak, R. (2013). Influence of traumatic acid on growth and metabolism of Chlorella vulgaris under conditions of salt stress. *Plant Growth Regulation* 73(2)**,** 103-110. doi: 10.1007/s10725-013-9872-x.

Qiu, X.M., Sun, Y.Y., Ye, X.Y., and Li, Z.G. (2019). Signaling Role of Glutamate in Plants. *Front Plant Sci* 10**,** 1743. doi: 10.3389/fpls.2019.01743.

Ramadan, K.M.A., Alharbi, M.M., Alenzi, A.M., El-Beltagi, H.S., Darwish, D.B.E., Aldaej, M.I., et al. (2022). Alpha Lipoic Acid as a Protective Mediator for Regulating the Defensive Responses of Wheat Plants against Sodic Alkaline Stress: Physiological, Biochemical and Molecular Aspects. *Plants (Basel)* 11(6). doi: 10.3390/plants11060787.

Rocchetti, G., Bernardo, L., Pateiro, M., Barba, F.J., Munekata, P.E.S., Trevisan, M., et al. (2020). Impact of a Pitanga Leaf Extract to Prevent Lipid Oxidation Processes during Shelf Life of Packaged Pork Burgers: An Untargeted Metabolomic Approach. *Foods* 9(11). doi: 10.3390/foods9111668.

Ruan, J., Zhou, Y., Zhou, M., Yan, J., Khurshid, M., Weng, W., et al. (2019). Jasmonic Acid Signaling Pathway in Plants. *Int J Mol Sci* 20(10). doi: 10.3390/ijms20102479.

Schenck, C.A., and Maeda, H.A. (2018). Tyrosine biosynthesis, metabolism, and catabolism in plants. *Phytochemistry* 149**,** 82-102. doi: 10.1016/j.phytochem.2018.02.003.

Schwachtje, J., Fischer, A., Erban, A., and Kopka, J. (2018). Primed primary metabolism in systemic leaves: a functional systems analysis. *Sci Rep* 8(1)**,** 216. doi: 10.1038/s41598-017-18397-5.

Shen, M., Li, J., Dong, Y., Liu, H., Peng, J., Hu, Y., et al. (2021). Profiling of Plant Growth-Promoting Metabolites by Phosphate-Solubilizing Bacteria in Maize Rhizosphere. *Plants (Basel)* 10(6). doi: 10.3390/plants10061071.

Shields, A., Shivnauth, V., and Castroverde, C.D.M. (2022). Salicylic Acid and N-Hydroxypipecolic Acid at the Fulcrum of the Plant Immunity-Growth Equilibrium. *Frontiers in Plant Science* 13. doi: 10.3389/fpls.2022.841688.

Sidiq, Y., Nakano, M., Mori, Y., Yaeno, T., Kimura, M., and Nishiuchi, T. (2021). Nicotinamide Effectively Suppresses Fusarium Head Blight in Wheat Plants. *Int J Mol Sci* 22(6). doi: 10.3390/ijms22062968.

Singh, P., Arif, Y., Miszczuk, E., Bajguz, A., and Hayat, S. (2022). Specific Roles of Lipoxygenases in Development and Responses to Stress in Plants. *Plants (Basel)* 11(7). doi: 10.3390/plants11070979.

Sirikantaramas, S., and Taura, F. (2017). "Cannabinoids: Biosynthesis and Biotechnological Applications," in *Cannabis sativa L. - Botany and Biotechnology*.), 183-206.

Szelenyi, M.O., Erdei, A.L., Josvai, J.K., Radvanyi, D., Sumegi, B., Vetek, G., et al. (2020). Essential Oil Headspace Volatiles Prevent Invasive Box Tree Moth (Cydalima perspectalis) Oviposition-Insights from Electrophysiology and Behaviour. *Insects* 11(8). doi: 10.3390/insects11080465.

Tani, A., Muramatsu, K., and Mochizuki, T. (2020). Emission of Methyl Ethyl Ketone and 2-Butanol Converted from Methyl Vinyl Ketone in Plant Leaves. *Atmosphere* 11(8). doi: 10.3390/atmos11080793.

Textor, S., Bartram, S., Kroymann, J., Falk, K.L., Hick, A., Pickett, J.A., et al. (2004). Biosynthesis of methionine-derived glucosinolates in Arabidopsis thaliana: recombinant expression and characterization of methylthioalkylmalate synthase, the condensing enzyme of the chain-elongation cycle. *Planta* 218(6)**,** 1026-1035. doi: 10.1007/s00425-003-1184-3.

Valette, M., Rey, M., Gerin, F., Comte, G., and Wisniewski-Dye, F. (2020). A common metabolomic signature is observed upon inoculation of rice roots with various rhizobacteria. *J Integr Plant Biol* 62(2)**,** 228-246. doi: 10.1111/jipb.12810.

Viana, V.E., Aranha, B.C., Busanello, C., Maltzahn, L.E., Panozzo, L.E., de Oliveira, A.C., et al. (2022). Metabolic profile of canola (Brassica napus L.) seedlings under hydric, osmotic and temperature stresses. *Plant Stress* 3. doi: 10.1016/j.stress.2022.100059.

Volz, R., Park, J.Y., Harris, W., Hwang, S., and Lee, Y.H. (2021). Lyso-phosphatidylethanolamine primes the plant immune system and promotes basal resistance against hemibiotrophic pathogens. *BMC Biotechnol* 21(1)**,** 12. doi: 10.1186/s12896-020-00661-8.

Wang, Y., Liu, J., Yang, F., Zhou, W., Mao, S., Lin, J., et al. (2021). Untargeted LC-MS-based metabolomics revealed specific metabolic changes in cotyledons and roots of Ricinus communis during early seedling establishment under salt stress. *Plant Physiol Biochem* 163**,** 108-118. doi: 10.1016/j.plaphy.2021.03.019.

War, A.R., Paulraj, M.G., Ahmad, T., Buhroo, A.A., Hussain, B., Ignacimuthu, S., et al. (2012). Mechanisms of plant defense against insect herbivores. *Plant Signal Behav* 7(10)**,** 1306-1320. doi: 10.4161/psb.21663.

Wasternack, C. (2007). Jasmonates: an update on biosynthesis, signal transduction and action in plant stress response, growth and development. *Ann Bot* 100(4)**,** 681-697. doi: 10.1093/aob/mcm079.

Wu, Y., Liao, W., Dawuda, M.M., Hu, L., and Yu, J. (2018). 5-Aminolevulinic acid (ALA) biosynthetic and metabolic pathways and its role in higher plants: a review. *Plant Growth Regulation* 87(2)**,** 357-374. doi: 10.1007/s10725-018-0463-8.

Xiao, X., Erukainure, O.L., Sanni, O., Koorbanally, N.A., and Islam, M.S. (2020). Phytochemical properties of black tea (Camellia sinensis) and rooibos tea (Aspalathus linearis); and their modulatory effects on key hyperglycaemic processes and oxidative stress. *J Food Sci Technol* 57(12)**,** 4345-4354. doi: 10.1007/s13197-020-04471-w.

Yang, C., Zhao, W., Wang, Y., Zhang, L., Huang, S., and Lin, J. (2020). Metabolomics Analysis Reveals the Alkali Tolerance Mechanism in Puccinellia tenuiflora Plants Inoculated with Arbuscular Mycorrhizal Fungi. *Microorganisms* 8(3). doi: 10.3390/microorganisms8030327.

Zandona, G.P., Bagatini, L., Woloszyn, N., de Souza Cardoso, J., Hoffmann, J.F., Moroni, L.S., et al. (2020). Extraction and characterization of phytochemical compounds from aracazeiro (Psidium cattleianum) leaf: Putative antioxidant and antimicrobial properties. *Food Res Int* 137**,** 109573. doi: 10.1016/j.foodres.2020.109573.

Zeier, J. (2013). New insights into the regulation of plant immunity by amino acid metabolic pathways. *Plant Cell Environ* 36(12)**,** 2085-2103. doi: 10.1111/pce.12122.

Zhang, W., Qin, W., Li, H., and Wu, A.M. (2021). Biosynthesis and Transport of Nucleotide Sugars for Plant Hemicellulose. *Front Plant Sci* 12**,** 723128. doi: 10.3389/fpls.2021.723128.

Yasuno, R., & Wada, H. (1998). Biosynthesis of lipoic acid in Arabidopsis: cloning and characterization of the cDNA for lipoic acid synthase. *Plant physiology*, *118*(3), 935–943. https://doi.org/10.1104/pp.118.3.935
